# A Novel Ubiquitin Ligase Adaptor PTPRN Suppresses Seizure Susceptibility through Endocytosis of Na_V_1.2 Sodium Channels

**DOI:** 10.1002/advs.202400560

**Published:** 2024-06-14

**Authors:** Yifan Wang, Hui Yang, Na Li, Lili Wang, Chang Guo, Weining Ma, Shiqi Liu, Chao Peng, Jiexin Chen, Huifang Song, Hedan Chen, Xinyue Ma, Jingyun Yi, Jingjing Lian, Weikaixin Kong, Jie Dong, Xinyu Tu, Mala Shah, Xin Tian, Zhuo Huang

**Affiliations:** ^1^ State Key Laboratory of Natural and Biomimetic Drugs Department of Molecular and Cellular Pharmacology School of Pharmaceutical Sciences Peking University Beijing 100191 China; ^2^ IDG/McGovern Institute for Brain Research Peking University Beijing 100871 China; ^3^ Department of Neurology Shengjing Hospital Affiliated to China Medical University Shenyang 110022 China; ^4^ UCL School of Pharmacy University College London London WC1N 1AX UK; ^5^ Department of Neurology The First Affiliated Hospital of Chongqing Medical University Chongqing Key Laboratory of Neurology Chongqing 400016 China

**Keywords:** endocytosis, epilepsy, intrinsic plasticity, Na_V_1.2, NEDD4L, PTPRN

## Abstract

Intrinsic plasticity, a fundamental process enabling neurons to modify their intrinsic properties, plays a crucial role in shaping neuronal input‐output function and is implicated in various neurological and psychiatric disorders. Despite its importance, the underlying molecular mechanisms of intrinsic plasticity remain poorly understood. In this study, a new ubiquitin ligase adaptor, protein tyrosine phosphatase receptor type N (PTPRN), is identified as a regulator of intrinsic neuronal excitability in the context of temporal lobe epilepsy. PTPRN recruits the NEDD4 Like E3 Ubiquitin Protein Ligase (NEDD4L) to Na_V_1.2 sodium channels, facilitating NEDD4L‐mediated ubiquitination, and endocytosis of Na_V_1.2. Knockout of PTPRN in hippocampal granule cells leads to augmented Na_V_1.2‐mediated sodium currents and higher intrinsic excitability, resulting in increased seizure susceptibility in transgenic mice. Conversely, adeno‐associated virus‐mediated delivery of PTPRN in the dentate gyrus region decreases intrinsic excitability and reduces seizure susceptibility. Moreover, the present findings indicate that PTPRN exerts a selective modulation effect on voltage‐gated sodium channels. Collectively, PTPRN plays a significant role in regulating intrinsic excitability and seizure susceptibility, suggesting a potential strategy for precise modulation of Na_V_1.2 channels' function.

## Graphical abstract

Intrinsic plasticity, crucial for neurons adapting to environmental changes, plays an essential role in many neurological disorders including epilepsy. The protein tyrosine phosphatase receptor type N (PTPRN) is identified as a novel regulator of intrinsic plasticity. In response to aberrant neuronal activity, PTPRN expression escalates, promoting the endocytosis of Na_V_1.2 channels, to balance aberrant neuronal excitability and reduce seizure susceptibility.

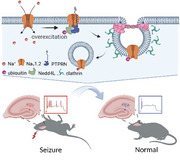

## 1 Introduction

Neuronal plasticity is a fundamental property of the nervous system that enables rapid adaptation in response to changes in an organism's internal and external environment.^[^ ^1^ ^]^ This lifelong process mediates the structural and functional modifications of dendrites, axons, somata, and synapses and can be categorized into two types: synaptic and intrinsic. Synaptic plasticity refines neuronal networks by strengthening, weakening, pruning, and adding synaptic connections,^[^ ^2^ ^]^ while intrinsic plasticity alters the intrinsic membrane excitability of postsynaptic neurons, modifying the input‐output information flow from dendrites to axon terminals,^[^ ^3^ ^]^ by changing the number, distribution, and activity of various ion channels within the neuron.^[^ ^4^ ^]^

Epilepsy is a neurological disorder characterized by abnormal and excessive neuronal activity in the brain,^[^ ^5^ ^]^ with intrinsic plasticity mechanisms playing a crucial role in both the generation and propagation of epileptic activity,^[^ ^6^ ^]^ as well as the inhibition of epileptogenesis.^[^ ^7^ ^]^ Alterations in ion channel expression and distribution contribute to the development and maintenance of epilepsy, while compensatory mechanisms help limit the spread of seizures and prevent the progression of epilepsy. These complex interactions between intrinsic plasticity and epilepsy underscore the need to better understand the mechanisms involved in epileptogenesis. Although considerable progress has been made in understanding the transcriptional tuning of ion channels,^[^ ^6^, ^8^ ^]^ which enables long‐lasting sculpting of the neurons, the mechanisms underlying ion channel trafficking in the context of intrinsic plasticity and epileptogenesis remain poorly understood, highlighting the need for further research.

The voltage‐gated sodium channel isoform 1.2 (Na_V_1.2), encoded by *SCN2A*, is a major type of sodium channel expressed in the neurons of the central nervous system.^[^ ^9^ ^]^ Na_V_1.2 plays a crucial role in the initiation and propagation of action potentials in neurons,^[^ ^10^ ^]^ and in this way determines the excitability of neurons. Dysfunctional Na_V_1.2 channel activity due to mutations in *SCN2A* has been linked to a range of neurological disorders, including infantile epileptic encephalopathy, benign (familial) infantile seizures, and autism spectrum disorder/intellectual disability.^[^ ^10a^ ^]^ Specifically, gain‐of‐function (GOF) mutations of *SCN2A* are strongly associated with the development of epilepsy,^[^ ^10^, ^11^ ^]^ as they result in enhanced Na_V_1.2 channel activity, leading to a hyperexcitable state in neurons and increasing the onset frequency of seizures. Investigating the regulation of Na_V_1.2 channel function is thus a crucial area of research with potential therapeutic implications for epilepsy.

Recent advancements in microarray and high‐throughput sequencing technologies have facilitated the use of bioinformatics methods to uncover molecular mechanisms that underlie complex neurological disorders. In this study, we employed weighted gene co‐expression network analysis (WGCNA) and identified the protein tyrosine phosphatase receptor type N (PTPRN) as a regulator of intrinsic neuronal excitability in temporal lobe epilepsy (TLE) patients and TLE rodent models. We discovered that enhanced neuronal activity led to increased expression of PTPRN, which selectively downregulated Na_V_1.2 function by recruiting the NEDD4 Like E3 Ubiquitin Protein Ligase (NEDD4L) to these channels for ubiquitination and endocytosis, thereby compensating for aberrant neuronal excitability and seizure susceptibility. Our findings provide insight into the distinctive regulatory mechanism of Na_V_1.2 and establish a molecular link between neuronal activity and ion channel trafficking.

## 2 Results

### 2.1 Patients and Rodents with Temporal Lobe Epilepsy have Aberrantly High PTPRN Expression Levels

**Figure 1:**
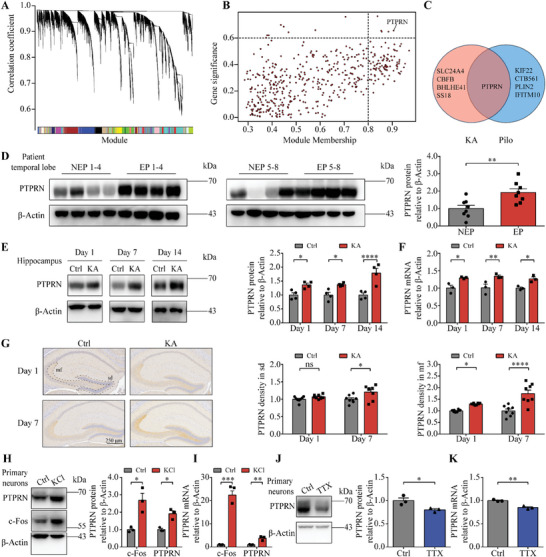
Patients and rodents with temporal lobe epilepsy have aberrantly high PTPRN levels. A) Identification of modules in the kainic acid (KA) model. A total of 25 modules were identified in a hierarchical clustering tree diagram. B) Scatterplot of gene significance versus module membership for genes in the most relevant module. Candidate critical genes were selected according to thresholds: gene significance > 0.6 and module membership > 0.8 (dotted line). C) Venn diagram showing the overlap between candidate critical genes identified from KA model data and pilocarpine (Pilo) model data. D) Left panel: Western blot analysis of PTPRN in the temporal lobe tissues from non‐epilepsy patients (NEP) or temporal lobe epilepsy patients (EP). Right panel: Quantification of the results by normalizing the protein levels of PTPRN to those of β‐Actin. *n* = 8, **p < 0.01, unpaired two‐tailed Student's t‐test. E) Left panel: Western blot analysis of PTPRN in hippocampal tissues obtained from mice injected with KA or saline (Ctrl) at 1, 7, and 14 days after injection. Right panel: Quantification of the results by normalizing the protein levels of PTPRN to those of β‐Actin. *n* = 4 mice in each group, *p < 0.05, ****p < 0.0001, two‐way ANOVA with Bonferroni's multiple‐comparisons test. F) RT‐PCR analysis of PTPRN in hippocampal tissues obtained from mice injected with KA or saline (Ctrl) at 1, 7, and 14 days after injection. The levels of mRNA were normalized to those of β‐Actin. *n* = 4 mice in each group, *p < 0.05, **p < 0.01, two‐way ANOVA with Bonferroni's multiple‐comparisons test. G) Left panel: Immunohistochemistry images showing the expression of PTPRN in the hippocampus from mice injected with KA (KA) or saline (Ctrl) respectively at 1 and 7 days after injection. Scale bar, 250 µm. mf, mossy fiber; sd, somatodendritic compartment. Middle panel: Quantification of PTPRN protein in sd. Right panel: Quantification of PTPRN protein in mf. *n* = 8 mice in each group, *p < 0.05, **p < 0.01, ****p < 0.0001, two‐way ANOVA with Bonferroni's multiple‐comparisons test. H) Left panel: Western blot analysis of indicated proteins in cultured primary cortical neurons treated with vehicle (control) or 50 mm KCl for 24 h. Right panel: Quantification of the results by normalizing the protein levels of indicated genes to those of β‐Actin. *n* = 3, *p < 0.05, two‐way ANOVA with Bonferroni's multiple‐comparisons test. I) RT‐PCR analysis of indicated genes in cultured primary cortical neurons described in (**H**). The levels of mRNA were normalized to those of β‐Actin. *n* = 3, **p < 0.01, ***p < 0.001, two‐way ANOVA with Bonferroni's multiple‐comparisons test. J) Left panel: Western blot analysis of PTPRN in cultured primary cortical neurons treated with vehicle (control) or 1 µm TTX for 24 hours. Right Panel: Quantification of the results by normalizing PTPRN protein levels to those of β‐Actin. *n* = 3, *p < 0.05, unpaired two‐tailed Student's t‐test. K) RT‐PCR analysis of PTPRN in cultured primary cortical neurons described in (J). The levels of mRNA were normalized to those of β‐Actin. *n* = 3, **p < 0.01, unpaired two‐tailed Student's t‐test. Data are represented as mean ± s.e.m.

To increase the understanding of the mechanisms underlying temporal lobe epilepsy (TLE), we conducted WGCNA using RNA sequencing data from rat TLE models (GSE47752) in a hypothesis‐free manner^[^ ^12^ ^]^ to screen for genes critical for drug‐induced epilepsy. First, we analyzed data from the kainic acid (KA) model, using a total of 24 raw files (6 control samples and 18 epilepsy samples) derived from rat hippocampal granule cells to construct the gene co‐expression network. Twenty‐five modules were identified after hierarchical clustering and red module showed the most significant correlation with epilepsy phenotype (Pearson correlation coefficient = 0.47, *p* = 0.02) (**Figure** **1**A; and Table S1, Supporting Information). To identify critical genes in this module, we plotted the gene significance against module membership and identified five candidate critical genes that met our criteria (gene significance > 0.6, module membership > 0.8) (Figure 1B). We additionally analyzed raw files from the pilocarpine model in the same way (Figure S1A,B and Table S2, Supporting Information), and the only candidate critical gene found in both TLE models was PTPRN (Figure 1C). PTPRN belongs to the family of receptor‐type protein tyrosine phosphatases (RPTPs) and has been identified as a major autoantigen in type 1 diabetes.^[^ ^13^ ^]^ While extensive research has elucidated its role in regulating insulin secretion and glucose homeostasis in pancreatic β‐cells,^[^ ^14^ ^]^ the broad expression of PTPRN suggests that it may have wider functions.^[^ ^15^ ^]^ In particular, the role of PTPRN in hippocampal granule cells remains poorly understood, making it an intriguing area for further investigation in the context of TLE.

To further explore the potential involvement of PTPRN in TLE, we subsequently assessed its expression in temporal lobe tissues surgically removed from patients (Table S3, Supporting Information). Our analysis revealed significantly higher PTPRN protein levels in samples from TLE patients compared with those without the condition (Figure 1D). We also observed enhanced PTPRN expression in two animal models of epilepsy. In the hippocampal tissues from mice, KA injection led to an increase of PTPRN protein levels 1–14 days post‐treatment, as demonstrated by western blot analysis (Figure 1E). Real‐time quantitative reverse transcriptase PCR (RT‐PCR) analysis also confirmed the upregulation of PTPRN mRNA levels (Figure 1F). In addition, we observed enhanced expression of PTPRN in hippocampal tissues from rats 1–14 days post pilocarpine injection (Figure S1C–E, Supporting Information). We next investigated PTPRN protein localization by performing immunohistochemical analysis and found that PTPRN expression was concentrated in the dentate gyrus (DG) and that expression increased in both the mossy fiber (mf) and somatodendritic compartment (sd) of the granule cells following KA administration (Figure 1G).

Because epilepsy is characterized by recurrent spontaneous seizures due to the hyperexcitability and hypersynchrony of brain neurons,^[^ ^16^ ^]^ we further investigated whether PTPRN expression responds to neural activities. In cultured primary cortical neurons from mouse pups, membrane depolarization induced by potassium chloride stimulation led to an increase of PTPRN mRNA and protein levels, coinciding with the induction of c‐Fos, which was included as a marker of recent neuronal activation^[^ ^17^ ^]^ (Figure 1H,I). On the contrary, inhibiting neuronal activity via pharmacological administration of the sodium channel blocker tetrodotoxin (TTX) reduced PTPRN expression (Figure 1J,K).^[^ ^18^ ^]^

Together, these results indicate that PTPRN expression is upregulated in response to aberrant neuronal activities, suggesting a potential role of PTPRN in neuronal plasticity in the context of TLE.

### 2.2 PTPRN Reduces Neuronal Intrinsic Excitability and Suppresses Seizure Susceptibility

**Figure 2:**
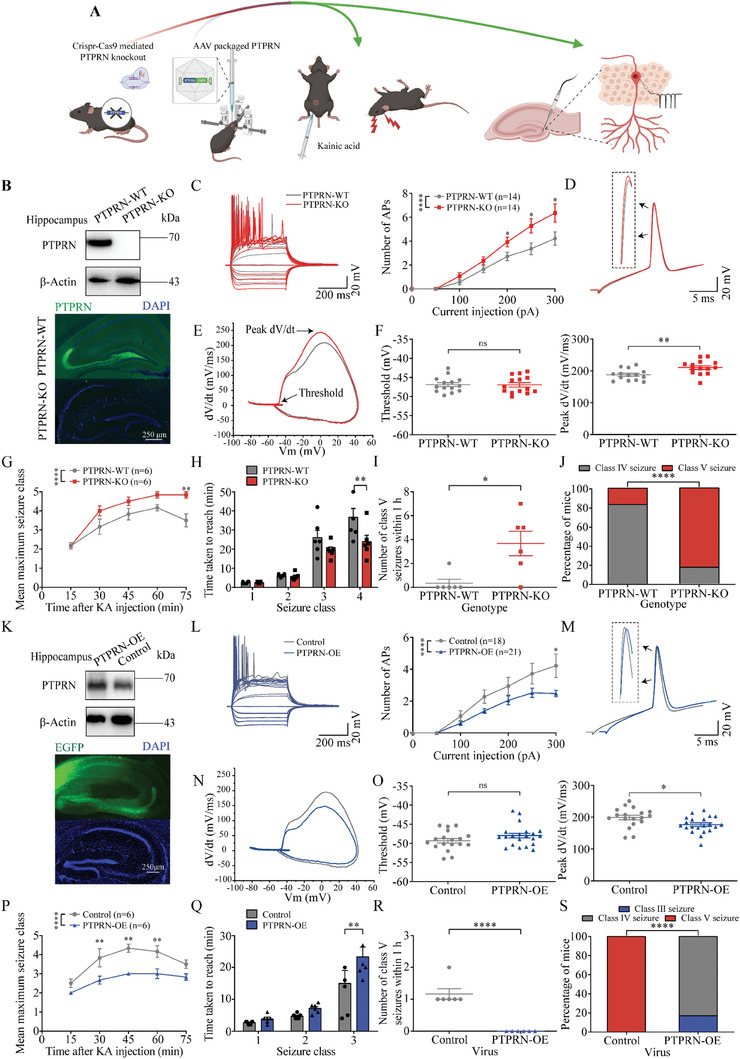
PTPRN reduces neuronal intrinsic excitability and suppresses seizure susceptibility. A) Illustration of the of the experimental procedures. Left penal: Schematic diagram showing the targeting strategy for generating PTPRN‐KO mice using the CRISPR‐Cas9 system (left) and stereotaxic injection of AAV expressing PTPRN‐T2A‐EGFP in the hippocampal DG region (right). Right panel: Schematic diagram showing seizure susceptibility assessment with KA model (left) and neuronal excitability evaluated by whole‐cell current‐clamp recordings on DG granule cells (right). B) Top panel: validation of PTPRN‐KO mice by western blot analysis using hippocampal tissues. Bottom panel: Staining for PTPRN in hippocampi of adult PTPRN‐KO and PTPRN‐WT mice. Scale bar, 250 µm. C) Left panel: Representative current‐clamp recordings obtained from DG granule cells in PTPRN‐KO and PTPRN‐WT mice. A series of 400 ms hyperpolarizing and depolarizing steps in 50 pA increments were applied to produce the traces. For comparison, the cells were held at −80 mV of membrane potential. Right panel: The mean number of APs generated in response to depolarizing current pulses. *p < 0.05, ****p < 0.0001 two‐way ANOVA with Bonferroni's multiple‐comparisons test. D) Typical APs from DG granule cells in PTPRN‐KO and PTPRN‐WT mice. E) Phase plane plots associated with APs in (D). F) The average AP threshold (left) and peak rising phase dV/dt (right). **p < 0.01, unpaired two‐tailed Student's t‐test. G) Graph depicting the seizure progression in PTPRN‐KO and PTPRN‐WT mice, illustrated as mean maximum seizure class reached by 15, 30, 45, 60, and 75 min after KA administration. *n* = 6, **p < 0.01, ****p < 0.0001, two‐way ANOVA with Bonferroni's multiple‐comparisons test. H) Graph showing time taken to reach each class of seizure after administration of KA in PTPRN‐KO and PTPRN‐WT mice. *n* = 6, **p < 0.01, two‐way ANOVA with Bonferroni's multiple‐comparisons test. I) Number of class V seizures within 1 h after KA administration. *n* = 6, *p < 0.05, unpaired two‐tailed Student's t‐test. J) Incidence of maximum seizure class reached during the course of the experiments in (**G**). *n* = 6, ****p < 0.0001, Chi‐square test. K) Top panel: Validation of PTPRN delivery by western blot analysis using hippocampal tissues from mice infected with control AAV (control) or AAV expressing PTPRN (PTPRN‐OE) for 14 days. Bottom panel: Infection of AAV expressing PTPRN‐T2A‐EGFP in the hippocampal DG region 14 days after injection. Scale bar, 250 µm. L) Left panel: Representative current‐clamp recordings obtained from DG granule cells in PTPRN‐OE and control mice. A series of 400 ms hyperpolarizing and depolarizing steps in 50 pA increments were applied to produce the traces. For comparison, the cells were held at −80 mV of membrane potential. Right panel: The mean number of APs generated in response to depolarizing current pulses. *p < 0.05, ****p < 0.0001, two‐way ANOVA with Bonferroni's multiple‐comparisons test. M) Typical APs obtained from DG granule cells in PTPRN‐OE and control mice. N) Phase plane plots associated with APs in (M). O) The average AP threshold (left) and peak rising phase dV/dt (right). *p < 0.05, unpaired two‐tailed Student's t‐test. P) Graph depicting the seizure progression in PTPRN‐OE and control mice, illustrated as mean maximum seizure class reached by 15, 30, 45, 60, and 75 min after KA administration. *n* = 6, **p < 0.01, ****p < 0.0001, two‐way ANOVA with Bonferroni's multiple‐comparisons test. Q) Graph showing time taken to reach each class of seizure after administration of KA in PTPRN‐OE and control mice. *n* = 6, **p < 0.01, two‐way ANOVA with Bonferroni's multiple‐comparisons test. R) Number of class V seizures within 1 h after KA administration. *n* = 6, ****p < 0.0001, unpaired two‐tailed Student's t‐test. S) Incidence of maximum seizure class reached during the course of the experiments in (P). *n* = 6, ****p < 0.0001, Chi‐square test. Data are represented as mean ± s.e.m.

To investigate the pathophysiological consequences of aberrant PTPRN expression in vivo, we examined whether PTPRN expression affects seizure susceptibility in animal TLE models. We first generated PTPRN‐knockout (PTPRN‐KO) mice (**Figure** **2**A),^[^ ^19^ ^]^ in which complete elimination of PTPRN protein expression was verified by western blot analysis (Figure 2B). PTPRN‐KO mice appeared grossly normal compared with their wild‐type (PTPRN‐WT) littermates, with no significant difference in body weight. We then explored whether PTPRN modulates neuronal excitability, we performed whole‐cell current‐clamp recordings in the hippocampus of adult (6‐ to 8‐week‐old) mice while blocking excitatory and inhibitory inputs to the neurons. In view of the important role played by DG granule cells in promoting hyperexcitability in the context of epileptogenesis,^[^ ^20^ ^]^ we determined whether deletion of PTPRN had any effect on the intrinsic excitability of these cells in brain slices, which were identified by their large input resistance and complex dendritic trees. We observed a significantly increased number of action potentials (APs) in granule cells of PTPRN‐KO mice upon application of depolarizing current pulses, when membrane potentials were held at −80 mV (Figure 2C). Although the AP threshold was consistent between different genotypes (Figure 2D–F; and Table S4, Supporting Information), a closer examination of the AP waveform showed a pronounced elevation in the velocity of the APs (mV/ms) in PTPRN‐KO mice. Plotting AP velocity as a function of voltage (phase‐plane) reflected the recruitment of somatic sodium channels (Figure 2E).^[^ ^21^ ^]^ In DG granule cells of PTPRN‐KO mice, peak rising phase dV/dt, the somatic component of the AP was faster, indicating a more robust voltage‐gated sodium currents (I_Na_) in neuronal somatic compartments of PTPRN‐KO mice than that of PTPRN‐WT mice (Figure 2E; and Table S4, Supporting Information).

We further assessed seizure susceptibility with a commonly used KA model, in which seizures (evaluated with Racine scale) were induced in mice by administering KA intraperitoneally, followed by termination with the anticonvulsant sodium pentobarbital (SP).^[^ ^6^, ^22^ ^]^ After administering 25 mg kg^−1^ KA, PTPRN‐KO mice displayed significantly faster seizure progression, measured by maximum seizure class reached in each successive bin of 15 min (Figure 2G) and time taken to reach each seizure class (Figure 2H). Moreover, the seizure severity was considerably higher in PTPRN‐KO mice, with more of them developing class V seizures and more class V seizures generated, compared with PTPRN‐WT mice (Figure 2I,J).

Next, we performed gain‐of‐function experiments in which we injected adeno‐associated virus (AAV) expressing either PTPRN (PTPRN‐OE) or enhanced green fluorescent protein (EGFP, control) into the DG region of adult mice (Figure 2A). Two weeks after AAV injection, we confirmed the effectiveness of PTPRN delivery by conducting western blot analysis, which showed significantly higher PTPRN expression in virus‐infected neurons compared with control neurons (Figure 2K). We examined the intrinsic excitability of DG granule cells in brain slices. Although the AP threshold remained unchanged (Figure 2M–O; and Table S5, Supporting Information), we found reduced excitability in PTPRN‐OE mice, with fewer APs generated and lower AP velocity in the somatic component (Figure 2L,O; and Table S5, Supporting Information). We also assessed seizure susceptibility with 28 mg kg^−1^ KA, at which dose most WT mice could generate class V seizures. Our results demonstrated that AAV‐mediated PTPRN delivery in the DG region led to a lower seizure susceptibility, as observed in the comparison of PTPRN‐OE mice with the control group (Figure 2P–S).

The collective findings provide evidence that PTPRN reduces the intrinsic excitability of DG granule cells by reducing the somatic depolarizing current and suppresses seizure susceptibility.

**Figure 3:**
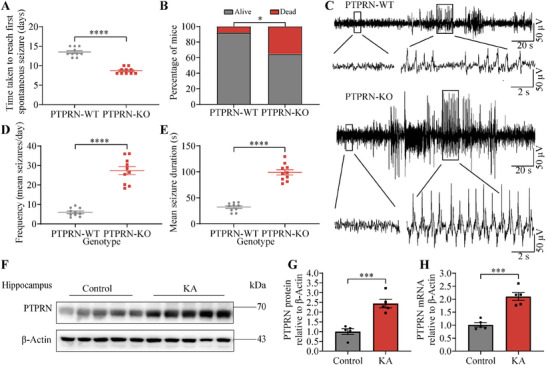
PTPRN suppresses seizure susceptibility and seizure severity in KA‐induced chronic seizure model. A) Time taken to reach first spontaneous seizure after administration of KA from PTPRN‐KO and PTPRN‐WT mice. *n*  =  10, ****p < 0.0001, unpaired two‐tailed Student's t‐test. B) Mortality of PTPRN‐KO mice (*n* = 17) and PTPRN‐WT (*n* = 26) mice from 48 h after SE termination to chronic phase of epilepsy. *p < 0.05, Chi‐square test. C) Examples of in vivo EEG recordings obtained from PTPRN‐KO and PTPRN‐WT mice. D) Quantification of convulsive seizure frequency. Convulsive seizure frequency was calculated as the mean number of convulsive seizures per day for each mouse. *n* = 10, ****p < 0.0001, unpaired two‐tailed Student's t‐test. E) Quantification of convulsive seizure duration. *n* = 10, ****p < 0.0001, unpaired two‐tailed Student's t‐test. F) Western blot analysis of PTPRN in hippocampal tissues obtained from mice four weeks post‐SE. G) Quantification of the results in (F) by normalizing the protein levels of PTPRN to those of β‐Actin. *n* = 5, ***p < 0.001, unpaired two‐tailed Student's t‐test. H) RT‐PCR analysis of PTPRN in hippocampal tissues obtained from mice described in (F). The levels of mRNA were normalized to those of β‐Actin. *n* = 5, ***p < 0.001, unpaired two‐tailed Student's t‐test. Data are represented as mean ± s.e.m.

To ascertain whether changes in PTPRN levels influenced the progression of chronic seizures, we generated a kainic acid (KA)‐induced chronic seizure model. Status epilepticus (SE) remitted after the administration of SP, and the first spontaneous seizures were generally observed between 5 and 30 days later, with an average of 14 days after SE termination.^[^ ^23^ ^]^ Spontaneous seizures were identified using electroencephalography (EEG) recordings and synchronous video monitoring after SE. Consistent with the normal course of epilepsy in this model, the latency between SE termination and the first spontaneous seizure averaged 13.5 days in PTPRN‐WT mice (13.55 ± 0.34 days; **Figure** **3**A). Notably, PTPRN‐KO mice exhibited significantly earlier seizure onset, occurring at 8.7 days post‐SE (8.7 ± 0.24 days; Figure 3A). In addition, PTPRN‐KO mice showed higher mortality in the latent period of epilepsy after induction of SE (Figure 3B). To measure changes in spontaneous seizure dynamics during the chronic phase of epilepsy, we quantified chronic spontaneous convulsive seizures during the 2–3 weeks post KA injection (Figure 3C–E; and Video S1, Supporting Information). EEG recordings and synchronous video monitoring revealed that the frequency and duration of convulsive seizure were increased in PTPRN‐KO mice (Figure 3D,E). Furthermore, we investigated alterations in PTPRN expression in chronic epilepsy. Western blot and RT‐PCR analysis demonstrated a significant upregulation of PTPRN protein levels in hippocampal tissues isolated from mice four weeks post‐SE (Figure 3F–H). These results further support the notion that PTPRN suppresses seizure susceptibility and seizure severity.

### 2.3 PTPRN Physically Interacts with Na_V_1.2 Sodium Channels and Negatively Modulates Na_V_1.2‐Mediated Currents

**Figure 4:**
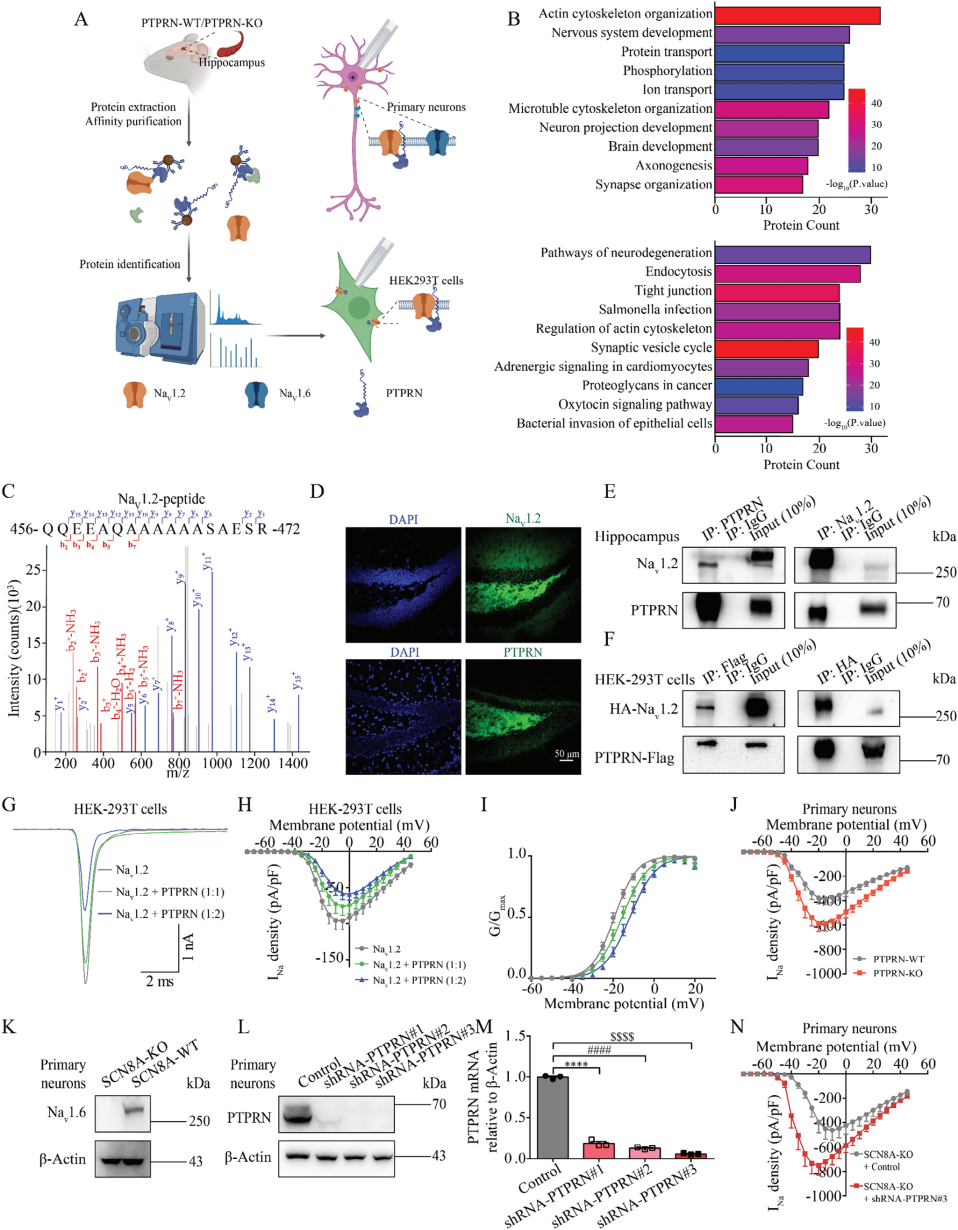
PTPRN interacts with Na_V_1.2 channels and negatively modulates Na_V_1.2‐mediated current. A) Workflow showing experimental procedures for identification of PTPRN interactors and whole‐cell patch‐clamp recordings. B) Top panel: Classification of the interactors identified in IP‐MS experiments with GO enrichment analysis. Bottom panel: Classification of the interactors identified in IP‐MS experiments with KEGG enrichment analysis. C) Tandem mass spectrometric spectra of a unique peptide of Na_V_1.2 identified in the IP‐PTPRN sample. D) Staining for Na_V_1.2 (top) and PTPRN (bottom) in hippocampal DG region of adult mice. Scale bar, 50 µm. E) Immunoblot analysis of Na_V_1.2 and PTPRN in IP‐PTPRN (left) and IP‐Na_V_1.2 (right) samples prepared from mouse hippocampal lysate. F) Immunoblot analysis of HA‐Na_V_1.2 and PTPRN‐Flag in IP‐Flag (Left) and IP‐HA (Right) samples prepared from HEK‐293T cell lysate. G) Representative whole‐cell currents recorded from HEK‐293T cells expressing Na_V_1.2 and empty vector (*n* = 18), Na_V_1.2 and PTPRN in a 1:1 ratio (*n* = 18), or Na_V_1.2 and PTPRN in a 1:2 ratio (*n* = 20). Currents were evoked by 5 mV steps depolarization from −90 to 45 mV and cells were held at ‐90 mV. H) Current density versus voltage relationship for the cells described in (G). Currents in all figures were normalized to cell capacitance. I) Graph depicting the voltage dependence of activation for Na_V_1.2 channels described in (G). The lines are the best‐fitted Boltzmann curves. J) Current density versus voltage relationship for the primary cortical neurons isolated from PTPRN‐KO (*n* = 13) or PTPRN‐WT mice (*n* = 16). Currents in all figures were normalized to cell capacitance. K) Validation of Scn8a deletion by western blot analysis using primary cortical neurons. L) Validation of PTPRN knockdown efficiency by western blot analysis using primary cortical neurons infected with lentivirus containing shRNA‐PTPRN or nonsilencing lentivirus for 7 days. M) RT‐PCR analysis of PTPRN in primary cortical neurons described in (L). The levels of mRNA were normalized to those of β‐Actin. *n* = 3, ****p < 0.0001, ####*p* < 0.0001, $$$$*p* < 0.0001, one‐way ANOVA with Bonferroni's multiple‐comparisons test. N) Current density versus voltage relationship for the primary cortical neurons isolated from Scn8a‐KO mice infected with lentivirus containing shRNA‐PTPRN#3 (*n* = 15) or nonsilencing lentivirus (*n* = 15) for 7 days. Currents in all figures were normalized to cell capacitance. Data are represented as mean ± s.e.m.

To decipher the molecular mechanisms by which PTPRN may inhibit intrinsic neuronal excitability and suppress seizure susceptibility, we performed immunoprecipitation‐mass spectrometry (IP‐MS) to identify proteins that interact with PTPRN. We used anti‐PTPRN antibody to enrich PTPRN and its interacting proteins from hippocampal lysates of adult PTPRN‐WT mice, with PTPRN‐KO mice used as a control against antibody off‐target effects^[^ ^24^ ^]^(**Figure** **4**A). To identify native PTPRN interactors, peptides from immunoprecipitation samples were analyzed and quantified using liquid chromatography tandem‐mass spectrometry. We applied thresholds for the number of unique peptides (≥ 4) and abundance ratio (≥ 4) to help in distinguishing between proteins that are putative constituents of the PTPRN complex, proteins that bound nonspecifically to the matrix, and potential false positives.^[^ ^25^ ^]^ Using this approach, we identified 354 coprecipitating proteins as PTPRN‐interacting proteins, among which PTPRN2, PTPRS, Dynamin‐1, PLP1, PDIA6, NCDN, HSPV5, CANX, and β‐Spectrin were previously reported to be part of the PTPRN interactome^[^ ^26^ ^]^ (Table S6, Supporting Information). Employing Gene Ontology (GO) enrichment analysis with a cutoff of ‐log_10_ *p* value ≥ 10, we found that PTPRN is potentially involved in cytoskeleton organization transport, nervous system development, and protein and ion transport (Figure 4B; and Table S7, Supporting Information); involvement in these processes was also suggested by Kyoto Encyclopedia of Genes and Genomes (KEGG) enrichment analysis (Figure 4B; and Table S8, Supporting Information).

Among the identified PTPRN interactors, we paid special attention to the Na_V_1.2 protein, which was annotated to the GO terms nervous system development as well as ion transport and was the only voltage‐gated sodium channel (Na_V_) discovered in our IP‐MS results. The Na_V_1.2 channel, which is encoded by *SCN2A*, supports neuronal excitability and is expressed primarily in excitatory neurons.^[^ ^27^ ^]^ Dysfunction of Na_V_1.2 channels has been identified as a leading cause of epilepsy in clinic.^[^ ^10^, ^28^ ^]^ In IP‐MS results, Na_V_1.2 was specifically captured from the hippocampal lysate of PTPRN‐WT mice (Figure 4C; and Table S6, Supporting Information). To explore whether Na_V_1.2 and PTPRN colocalize, we fixed and probed brain slices from adult mice with antibodies against Na_V_1.2 and PTPRN. As revealed by confocal analysis, Na_V_1.2 channels and PTPRN were colocalized in DG granule cells (Figure 4D). We also confirmed this interaction using immunoblot analysis, showing that Na_V_1.2 channels were detected by anti‐Na_V_1.2 antibody in the sample purified from mice hippocampus with anti‐PTPRN antibody, but not in a control sample obtained with a nonspecific rabbit IgG. We also immunopurified Na_V_1.2 channels from hippocampal lysates using anti‐Na_V_1.2 antibody and observed that PTPRN was present in the precipitation according to immunoblot analysis (Figure 4E). This interaction was further verified in a heterologous expression system. Specifically, we fused tandem Flag tags to the mature form PTPRN (aa 449–979), the major PTPRN species detected in tissue lysates, which was converted from pro‐PTPRN (Figure S2A, Supporting Information).^[^ ^29^ ^]^ We found that the immunoprecipitation of PTPRN‐Flag with anti‐Flag antibody simultaneously captured tandem HA‐tagged Na_V_1.2 channels in HEK‐293T cells, and vice versa (Figure 4F).

To examine whether PTPRN modulates Na_V_1.2 channel function, we transiently expressed PTPRN and Na_V_1.2 at different proportions in HEK‐293T cells and recorded the Na_V_1.2‐mediated currents using the whole‐cell patch‐clamp technique. Although PTPRN had no effect on Na_V_1.2 channel inactivation or recovery (Figure S2B,C and Table S9, Supporting Information), we found that the presence of PTPRN significantly reduced the voltage‐gated sodium currents (I_Na_) density (Figure 4G,H; and Table S9, Supporting Information), which was accompanied by a depolarizing shift of the activation curve (Figure 4I; and Table S9, Supporting Information), suggesting that PTPRN negatively modulates Na_V_1.2 channel function. Interestingly, PTPRN exhibited perfect subtype selectivity that it didn't modulate Na_V_1.5 or Na_V_1.6 channel function in HEK‐293T cells (Figure S2D–K and Tables S10 and S11, Supporting Information). To investigate whether PTPRN modulates the native Na_V_1.2‐mediated currents in vivo, we next performed whole‐cell patch‐clamp recordings in primary cortical neurons isolated from PTPRN‐KO and PTPRN‐WT mice. We observed remarkably higher I_Na_ density in neurons of PTPRN‐KO mice (Figure 4J; and Table S12, Supporting Information). As Na_V_ subtypes Na_V_1.2 and Na_V_1.6 are the two predominant forms in excitatory neurons,^[^ ^28^ ^]^ we subsequently recorded I_Na_ in primary cortical neurons isolated from C3HeB/FeJ Scn8a‐KO mice, in which the *Scn8a*‐encoded Na_V_1.6 channel was knocked out (Figure 4K). To this end, we designed three different PTPRN‐shRNAs to knock down PTPRN, and PTPRN‐shRNA#3 was selected because it had the highest knockdown efficiency (Figure 4L,M). Consistent with the aforementioned results, neurons infected with lentivirus expressing PTPRN‐shRNA#3 displayed a higher I_Na_ density compared with neurons infected with nonsilencing‐shRNA (control) (Figure 4N; and Table S13, Supporting Information), indicating PTPRN negatively modulates Na_V_1.2 channel function with subtype selectivity.

Together, these results demonstrate that PTPRN interacts with Na_V_1.2 channels and negatively modulates Na_V_1.2‐mediated currents both in vitro and in vivo.

### 2.4 PTPRN Promotes Ubiquitin‐Dependent Endocytosis of Na_V_1.2 Channels

**Figure 5:**
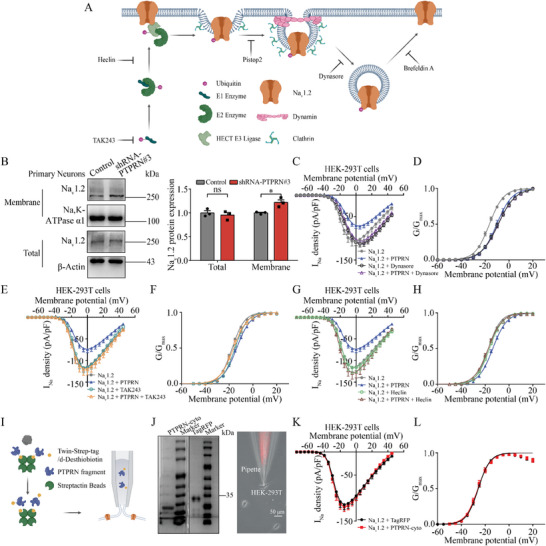
PTPRN promotes Na_V_1.2 channel internalization through ubiquitin‐dependent endocytosis. A) Schematic of the effect of inhibitors on the trafficking and ubiquitination of proteins. B) Left panel: Immunoblot analysis of cell surface biotinylation performed in primary cortical neurons infected with lentivirus containing shRNA‐PTPRN#3 or nonsilencing lentivirus for 7 days. Total lysates (total) and biotinylated fractions (membrane) were analyzed by western blot analysis. Right panel: Quantification of Na_V_1.2 total expression and surface expression. *n* = 3, *p < 0.05, two‐way ANOVA with Bonferroni's multiple‐comparisons test. C) Current density versus voltage relationship for HEK‐293T cells expressing Na_V_1.2 and PTPRN in a 1:2 ratio (Na_V_1.2 + PTPRN) or Na_V_1.2 and empty vector (Na_V_1.2), with or without treatment using dynasore (80 µm, 2 h). *n* = 21 for all samples except Na_V_1.2 + PTPRN plus dynasore (*n* = 24). D) Graph depicting voltage‐dependence of activation for Na_V_1.2 channels described in (C). The lines are the best‐fitted Boltzmann curves. E) Current density versus voltage relationship for HEK‐293T cells expressing Na_V_1.2 and PTPRN in a 1:2 ratio (Na_V_1.2 + PTPRN) or Na_V_1.2 and empty vector (Na_V_1.2), with or without treatment using TAK‐243 (1 µm, 2 h). *n* = 18 and 26 for untreated Na_V_1.2 and Na_V_1.2 + PTPRN, respectively, and *n* = 20 and 26 for Na_V_1.2 and Na_V_1.2 + PTPRN plus TAK‐243, respectively. F) Graph depicting voltage dependence of activation for Na_V_1.2 channels described in (E). The lines are the best‐fitted Boltzmann curves. G) Current density versus voltage relationship for HEK‐293T cells expressing Na_V_1.2 and PTPRN in a 1:2 ratio (Na_V_1.2 + PTPRN) or Na_V_1.2 and empty vector (Na_V_1.2), with or without treatment with Heclin (20 µm, 2 h). *n* = 18 and 26 for Na_V_1.2 and Na_V_1.2 + PTPRN plus Heclin, respectively, and *n* = 19 for both samples without treatment. H) Graph depicting voltage dependence of activation for Na_V_1.2 channels described in (G). The lines are the best‐fitted Boltzmann curves. I) Workflow showing experimental procedures for purification of PTPRN intracellular fragment (aa 601–979, PTPRN‐cyto) and TagRFP and whole cell patch‐clamp recordings. J) Left panel: Coomassie blue staining of PTPRN‐cyto (left) or TagRFP (right) after purification and separation by SDS‐PAGE. Right panel: Representative images showing peptide delivery through a pipette. Scale bar, 50 µm. K) Current density versus voltage relationship for HEK‐293T cells expressing Na_V_1.2 after intracellular equilibration with 1 µm PTPRN‐cyto (*n* = 25) or TagRFP (*n* = 23). L) Graph depicting voltage dependence of activation for Na_V_1.2 channels described in (K). The lines are the best‐fitted Boltzmann curves. Data are represented as mean ± s.e.m.

The strong increase of the I_Na_ density caused by knockout/knockdown of PTPRN, suggests that PTPRN may affect the expression or trafficking of Na_V_1.2 channels. To monitor Na_V_1.2 channel expression and trafficking, we performed a surface biotinylation assay in primary neurons infected with shRNA‐PTPRN#3 or nonsilencing‐shRNA. Despite the total level of Na_V_1.2 channels remaining the same, the surface expression of Na_V_1.2 channels significantly increased with knockdown of PTPRN (**Figure** **5**B), and consistent results were observed in the surface biotinylation assay performed in HEK‐293T cells (Figure S3A, Supporting Information). In addition, the finding in KEGG enrichment analysis that considerable PTPRN interactors are associated with endocytosis (Figure 4B; and Table S8, Supporting Information) suggested that PTPRN might modulate Na_V_1.2 channel function by promoting channel removal from the plasma membrane.

Pursuing this possibility, we treated HEK‐293T cells co‐expressing Na_V_1.2 and PTPRN with trafficking inhibitors and conducted whole‐cell patch‐clamp recording to study PTPRN's role in Na_V_1.2 channel modulation. Dynasore, a GTPase inhibitor that prevents endocytosis through inhibiting dynamin activity,^[^ ^30^ ^]^ was used first. We found that treatment with dynasore completely abolished the PTPRN‐mediated decrease in I_Na_ density (Figure 5C; and Table S14, Supporting Information), demonstrating that PTPRN modulates Na_V_1.2 channel localization via endocytosis. To exclude the possibility that PTPRN regulates intracellular Na_V_1.2 channel transport, we also used Brefeldin A (BFA) to block protein transport from the endoplasmic reticulum to the Golgi complex.^[^ ^31^ ^]^ Although BFA treatment significantly reduced I_Na_ density in HEK‐293T cells expressing Na_V_1.2 channels alone, it failed to eliminate PTPRN's inhibitory effect (Figure S3B and Table S15, Supporting Information). These results indicate that PTPRN does not influence intracellular transport. Instead, it re‐localizes Na_V_1.2 channels away from the membrane, leading to reduced I_Na_ densities. In general, clathrin‐mediated and ‐independent mechanisms have been the primary mechanisms identified for ion channel endocytosis.^[^ ^32^ ^]^ Because clathrin and adaptor‐related protein complex 2 (AP2), two critical components involved in clathrin‐mediated endocytosis (CME), were discovered as PTPRN interactors (Table S6, Supporting Information), we next asked whether PTPRN promotes Na_V_1.2 internalization via CME. Pitstop2, a clathrin assembly inhibitor,^[^ ^33^ ^]^ prevented the I_Na_ density reduction caused by PTPRN expression, suggesting the involvement of CME in Na_V_1.2 channel regulation by PTPRN (Figure S3D and Table S16, Supporting Information).

Additionally, we used ubiquitination inhibitors to interrogate the role of ubiquitination in this process because the ubiquitination of neuronal membrane proteins can regulate their internalization.^[^ ^34^ ^]^ TAK243 was selected because of its inhibitory effect on ubiquitin activating enzyme (E1 enzyme).^[^ ^35^ ^]^ With TAK243 treatment, there was a disappearance of reduction of I_Na_ density and the depolarizing shift of the activation curve induced by PTPRN (Figure 5E,F; and Table S17, Supporting Information). This phenomenon was consistent with that observed in HEK‐293T cells treated with Heclin, a HECT ubiquitin E3 ligase inhibitor.^[^ ^36^ ^]^ HECT family proteins are the ubiquitin E3 ligases reported to transfer ubiquitin to Na_V_ channels,^[^ ^37^ ^]^ and we found that the treatment with Heclin concurrently eliminated the I_Na_ density reduction and activation curve shift (Figure 5G,H; and Table S18, Supporting Information), suggesting that PTPRN changes the Na_V_1.2‐mediated current density and Na_V_1.2 channel gating property by promoting ubiquitin‐dependent endocytosis.

To further demonstrate that the negative shift of the Na_V_1.2 channel activation curve was the result of PTPRN‐induced long‐term ubiquitination, we designed an experiment to rule out the possibility that Na_V_1.2‐PTPRN physical interaction alone changes the activation of Na_V_1.2 channels. We purified the PTPRN intracellular fragment (aa 601–979, PTPRN‐cyto) and TagRFP (control) with a Twin‐Strep‐tag (Figure 5I), then delivered the purified proteins into HEK‐293T cells expressing Na_V_1.2 channels via patch pipettes (Figure 5J). Our results showed there was no change in the current density or activation curve (Figure 5K,L; and Table S19, Supporting Information), indicating that PTPRN regulates Na_V_1.2 channel biophysical properties through long‐term ubiquitination instead of transient interaction.

### 2.5 PTPRN Facilitates Ubiquitination of Na_V_1.2 Channels by Recruiting NEDD4L

**Figure 6:**
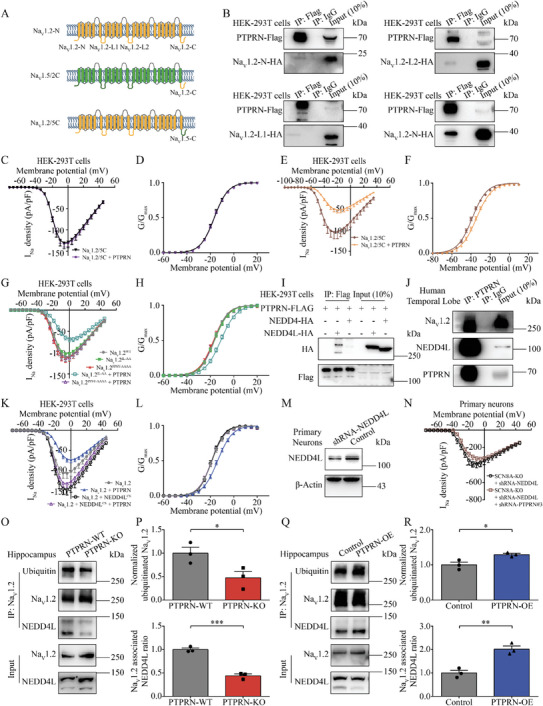
PTPRN facilitates ubiquitination of Na_V_1.2 channels by recruiting NEDD4L. A) Schematic diagram of Na_V_1.2 intracellular regions and Na_V_1.2‐1.5 chimeric channels (Na_V_1.2/5C and Na_V_1.5/2C). B) Immunoblot analysis of HA‐tagged Na_V_1.2 intracellular regions and PTPRN‐Flag in IP‐Flag samples prepared from HEK‐293T cell lysate. C) Current density versus voltage relationship for HEK‐293T cells expressing the Na_V_1.2/5C chimera and PTPRN in a 1:2 ratio (Na_V_1.2/5C + PTPRN) (*n* = 26) or the Na_V_1.2/5C chimera and empty vector in a 1:2 ratio (Na_V_1.2/5C, *n* = 19). D) Graph depicting voltage dependence of activation for the Na_V_1.2/5C chimera described in (C). The lines are the best‐fitted Boltzmann curves. E) Current density versus voltage relationship for HEK‐293T cells expressing the Na_V_1.5/2C chimera and PTPRN in a 1:2 ratio (Na_V_1.5/2C + PTPRN) or the Na_V_1.2/5C chimera and empty vector in a 1:2 ratio (Na_V_1.5/2C), *n* = 20. F) Graph depicting voltage‐dependence of activation for the Na_V_1.2/5C chimera described in (E). The lines are the best‐fitted Boltzmann curves. G) Current density versus voltage relationship for HEK‐293T cells expressing wild‐type (WT) Na_V_1.2, Na_V_1.2^IL‐AA^, or Na_V_1.2^PPSY‐AAAA^ mutations with PTPRN or empty vector in a 1:2 ratio. *n* = 20, 22, 23, 22, and 22 (from up to down in legend). H) Graph depicting voltage dependence of activation for Na_V_1.2 channels described in (G). The lines are the best‐fitted Boltzmann curves. I) Immunoblot analysis of HA‐tagged NEDD4/NEDD4L and PTPRN‐Flag in IP‐Flag samples prepared from HEK‐293T cell lysate. J) Immunoblot analysis of Na_V_1.2, NEDD4L and PTPRN in the IP‐PTPRN sample prepared from human temporal lobe lysate. K) Current density versus voltage relationship for HEK‐293T cells expressing WT Na_V_1.2 with or without PTPRN/NEDD4L^CS^ mutation. *n* = 18, 24, 24, and 23 (from up to down in legend). L) Graph depicting voltage dependence of activation for Na_V_1.2 channels described in (K). The lines are the best‐fitted Boltzmann curves. M) Validation of NEDD4L knockdown efficiency by western blot analysis using primary cortical neurons infected with lentivirus containing shRNA‐NEDD4L or nonsilencing lentivirus for 7 days. N) Current density versus voltage relationship for the primary cortical neurons isolated from Scn8a‐KO mice infected with lentivirus containing shRNA‐PTPRN#3 and shRNA‐NEDD4L (*n* = 15) or nonsilencing lentivirus (*n* = 15) for 7 days. Currents in all figures were normalized to cell capacitance. O) Western blot analysis of indicated proteins in IP‐Na_V_1.2 samples prepared from hippocampus of PTPRN‐KO or PTPRN‐WT mice. P) Quantification of Na_V_1.2 ubiquitination level (top) and the interaction between Na_V_1.2 and NEDD4L (bottom). *n* = 3, *p < 0.05, ***p < 0.001, unpaired two‐tailed Student's *t*‐test. Q) Western blot analysis of indicated proteins in IP‐Na_V_1.2 samples prepared from hippocampus of mice infected with control AAV or AAV expressing PTPRN. R) Quantification of Na_V_1.2 ubiquitination level (top) and the interaction between Na_V_1.2 and NEDD4L (bottom). *n* = 3, *p < 0.05, **p < 0.01, unpaired two‐tailed Student's *t*‐test. Data are represented as mean ± s.e.m.

To further elucidate the mechanism underlying Na_V_1.2 regulation by PTPRN, we performed co‐IP experiments to examine the regions of Na_V_1.2 protein involved in the interaction with PTPRN. We co‐expressed HA‐tagged intracellular fragments of Na_V_1.2 with Flag‐tagged PTPRN in HEK‐293T cells and found only the Na_V_1.2′s C‐terminus (Na_V_1.2‐C) was present in the sample purified from cell lysates with anti‐Flag antibody (**Figure** **6**B). To confirm that PTPRN's regulatory effect relies on the Na_V_1.2′s C‐terminus, we constructed two chimeras in which the C termini of the Na_V_1.2 and Na_V_1.5 channels were exchanged. (Figure 6A). We first recorded the function of the chimeras alone. Compared with WT Na_V_1.2, replacing the C‐terminal domain of Na_V_1.2 with that of Na_V_1.5 (Na_V_1.2/5C) positively shifted the steady‐state fast inactivation curve but had no effect on steady‐state activation curve (Figure S4A,B and Table S20, Supporting Information). Conversely, replacing the C‐terminal domain of Na_V_1.5 with that of Na_V_1.2 (Na_V_1.5/2C) negatively shifted the steady‐state fast inactivation curve, with steady‐state activation curve remaining unaffected (Figure S4A,B and Table S20, Supporting Information). These unique results were consistent with an earlier study,^[^ ^38^ ^]^ which indicated the successful construction of chimeras. Next, we tested PTPRN's effect on these chimeras and found that replacement with the Na_V_1.5′s C‐terminus endowed Na_V_1.2 with resistance to PTPRN modulation (Figure 6C,D; and Table S20, Supporting Information). On the contrary, fusion of Na_V_1.5 with the Na_V_1.2′s C‐terminus made Na_V_1.5 sensitive to the presence of PTPRN, which caused a change in activation and I_Na_ density (Figure 6E,F; and Table S20, Supporting Information).

We next searched for the motif in the Na_V_1.2′s C‐terminus responsive to PTPRN modulation. In the literature, the IL motif and PPSY motif are reported to play a role in the endocytosis of Na_V_1.2 channels. The IL motif (aa 1857–1858) governs Na_V_1.2 channel internalization via CME, possibly through its interaction with AP2,^[^ ^39^ ^]^ while the PPSY motif (aa 1972–1975) binds HECT E3 ligases, NEDD4 and NEDD4L, to initiate the ubiquitination process.^[^ ^40^ ^]^ To test whether PTPRN's regulatory effect relies on these motifs, we mutated the amino acids in these motifs into alanines respectively (Na_V_1.2^IL‐AA^ and Na_V_1.2^PPSY‐AAAA^). These changes did not impair the physical interaction between Na_V_1.2 and PTPRN (Figure S4C, Supporting Information). However, although the IL‐AA mutation had no effect on PTPRN modulation on Na_V_1.2, the replacement of the PPSY motif completely abolished PTPRN‐induced changes in both current density and the activation curve (Figure 6G,H; and Table S21, Supporting Information). Subsequent co‐IP experiments further verified that PTPRN physically interacts with NEDD4L in vitro and in vivo, but not with NEDD4, which was also supported by the identification of NEDD4L but not NEDD4 in IP‐MS (Figure 6I,J; and Table S6, Supporting Information). Together, these results suggest that PTPRN potentially acts as an adaptor to strengthen the interaction between E3 ligase NEDD4L and Na_V_1.2.

To test this hypothesis, we overexpressed a dominant negative mutant of NEDD4L, NEDD4L^CS^, which abolishes its catalytic activity,^[^ ^41^ ^]^ in HEK‐293T cells. We found that in the presence of the ligase‐deficient NEDD4L, additional expression of PTPRN did not affect Na_V_1.2 channel function (Figure 6K,L; and Table S22, Supporting Information). We also delivered shRNA targeting NEDD4L into primary neurons isolated from Scn8a‐KO mice, which significantly reduced NEDD4L expression (Figure 6M). Whole‐cell patch clamp recording revealed that knockdown of NEDD4L deprived PTPRN of its ability to regulate I_Na_ (Figure 6N; and Table S23, Supporting Information). Next, we measured the level of ubiquitinated Na_V_1.2 and quantified Na_V_1.2‐NEDD4L interaction in transgenic mice. We used anti‐Na_V_1.2 antibody to precipitate Na_V_1.2 protein from hippocampal lysates and analyzed the precipitates by western blot analysis. Immunoblot analysis with anti‐ubiquitin antibody showed a long band corresponding to ubiquitinated Na_V_1.2 (Figure S4D, Supporting Information). Although PTPRN had no effect on the total expression or tyrosine phosphorylation of the Na_V_1.2 protein (Figure S4E–I, Supporting Information), its deletion decreased the level of ubiquitinated Na_V_1.2 and reduced the interaction between NEDD4L and Na_V_1.2 (Figure 6O,P). Conversely, an increase in the levels of ubiquitinated Na_V_1.2 and the Na_V_1.2‐NEDD4L complex were observed after the AAV‐mediated delivery of PTPRN into the DG region (Figure 6Q,R). Together, these results demonstrate that PTPRN modulates Na_V_1.2 channel function by promoting NEDD4L‐mediated ubiquitination.

### 2.6 PTPRN‐Na_V_1.2 Axis Regulates Neuronal Intrinsic Excitability and Seizure Susceptibility

**Figure 7:**
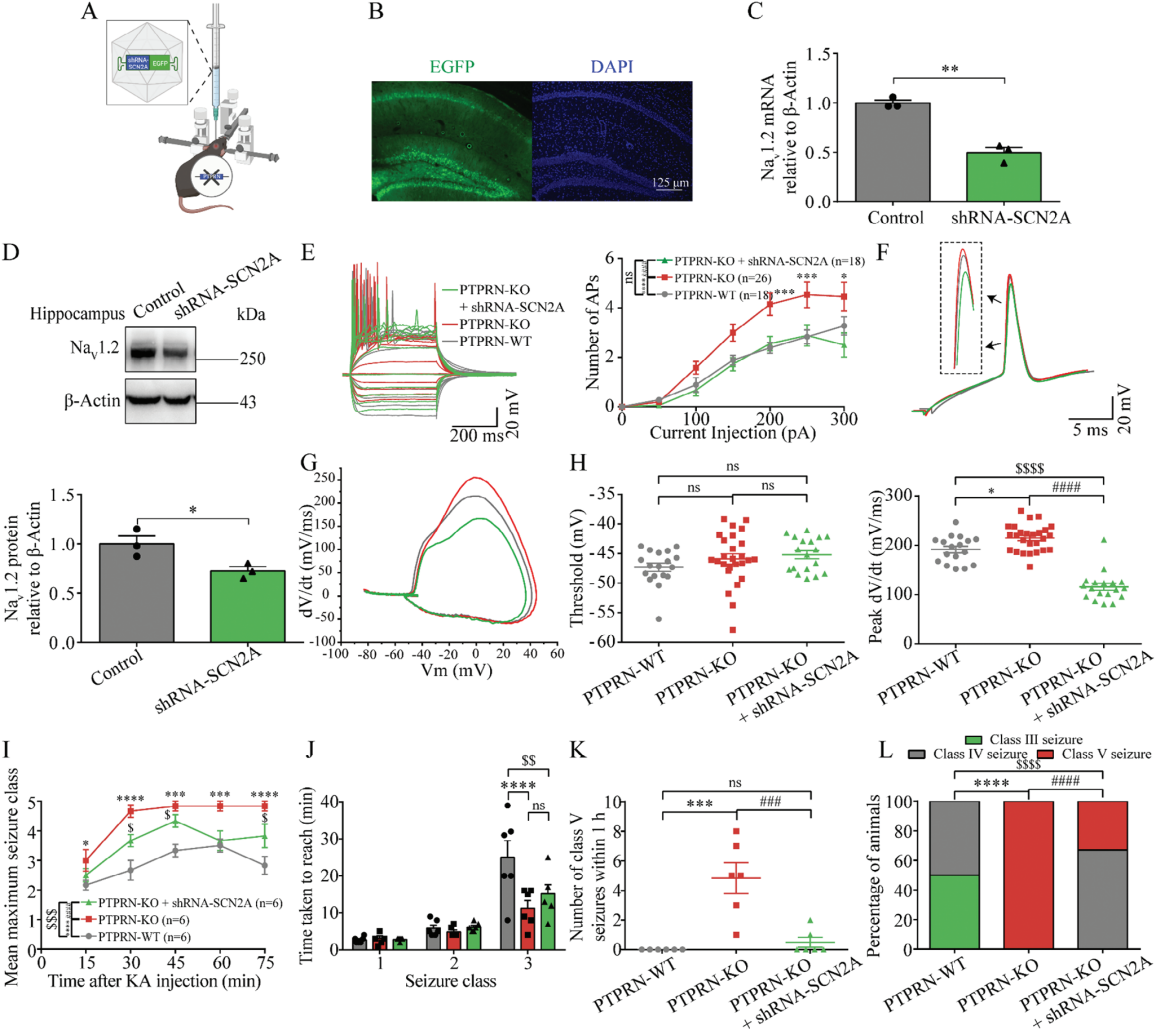
PTPRN‐Na_V_1.2 axis regulates neuronal intrinsic excitability and seizure susceptibility. A) Schematic diagram showing stereotaxic injection of shRNA‐Scn2a AAV in adult PTPRN‐KO mice. B) Infection of AAV expressing shRNA‐Scn2a and EGFP in the hippocampal DG region 14 days after injection. Scale bar, 125 µm. C) Validation of Scn2a knockdown efficiency by RT‐PCR analysis of Scn2a in hippocampal tissues obtained from mice infected with AAV‐containing shRNA‐Scn2a or control AAV for 14 days. The levels of mRNA were normalized to those of β‐Actin. *n* = 3, **p < 0.01, unpaired two‐tailed Student's *t*‐test. D) Top panel: Validation of Scn2a knockdown efficiency by western blot analysis using hippocampi from mice infected with AAV‐containing shRNA‐Scn2a or control AAV for 14 days. Bottom panel: Quantification of the results by normalizing the protein levels of Na_V_1.2 to those of β‐Actin. *n* = 3, *p < 0.05, unpaired two‐tailed Student's *t*‐test. E) Left panel: Representative current‐clamp recordings of DG granule cells from PTPRN‐WT and PTPRN‐KO mice infected with AAV containing shRNA‐Scn2a or control AAV for 14 days. For comparison, the cells were held at ‐80 mV of membrane potential. Right panel: The mean number of APs generated in response to depolarizing current pulses. *p < 0.05, ***p < 0.001, ****p < 0.0001, ####*p* < 0.0001, two‐way ANOVA with Bonferroni's multiple‐comparisons test. F) Typical APs of DG granule cells from mice described in (E). G) Phase plane plots associated with APs in (F). H) The average AP threshold (left) and peak rising phase dV/dt (right). *p < 0.05, ####*p* < 0.0001, $$$$*p* < 0.0001, one‐way ANOVA with Bonferroni's multiple‐comparisons test. I) Graph depicting the seizure progression in PTPRN‐WT and PTPRN‐KO mice infected with AAV containing shRNA‐Scn2a or control AAV for 14 days, illustrated as mean maximum seizure stage reached by 15, 30, 45, 60, and 75 min after KA administration. *n* = 6, $*p* < 0.05, *p < 0.05, ***p < 0.001, ****p < 0.0001, ###*p* < 0.001, $$$*p* < 0.001, two‐way ANOVA with Bonferroni's multiple‐comparisons test. J) Graph showing time taken to reach each stage of seizure after administration of KA in mice described in (I). *n* = 6, $$*p* < 0.01, ****p < 0.0001, two‐way ANOVA with Bonferroni's multiple‐comparisons test. K) Number of class V seizures within 1 h after KA administration. *n* = 6, ***p < 0.001, ###*p* < 0.001, one‐way ANOVA with Bonferroni's multiple‐comparisons test. L) Incidence of maximum seizure stage reached during the course of the experiments in (I). *n* = 6, ****p < 0.0001, ####*p* < 0.0001, $$$$*p* < 0.0001, Chi‐square test. Data are represented as mean ± s.e.m.

To confirm that altered Na_V_1.2 channel function underlies the aberrant intrinsic plasticity and seizure susceptibility mediated by PTPRN deletion, we sought to rescue the changes by reducing the expression of Na_V_1.2 in PTPRN‐KO mice (**Figure** **7**A). To this end, an AAV carrying shRNA‐Scn2a was generated and its effectiveness in silencing was confirmed (Figure 7B–D). While the deletion of PTPRN led to elevated intrinsic excitability of DG granule cells including increased AP numbers and somatic depolarization velocity, stereotaxic injection of shRNA‐Scn2a AAV reversed these changes (Figure 7E–H; and Table S24, Supporting Information). We also examined the effect of Scn2a knockdown on seizure susceptibility. After administrating of 25 mg kg^−1^ KA, the seizure severity was evaluated and we found that the delivery of shRNA‐Scn2a by AAV partially reversed the behavioral alteration in seizure susceptibility induced by PTPRN knockout (Figure 7I–L), confirming that PTPRN reduces seizure susceptibility through its negative regulation of Na_V_1.2 channel function.

Together, our study establishes a foundation for understanding PTPRN's influence on Na_V_1.2 channel function and lays the groundwork for further development of intervention methods for precision modulation of Na_V_1.2.

## 3 Discussion

**Figure 8:**
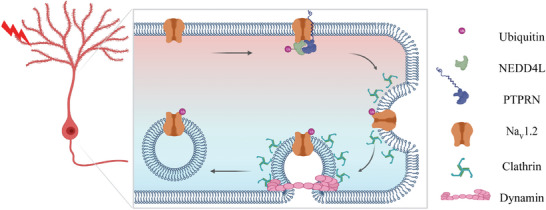
Proposed mechanism for PTPRN regulation of Na_V_1.2 channels and neuronal intrinsic plasticity. Elevated neuronal activity augments the recruitment of NEDD4L by PTPRN to Na_V_1.2 sodium channels. This interaction promotes NEDD4L‐mediated ubiquitination, subsequently leading to the endocytosis of Na_V_1.2 channels. The presented regulatory mechanism provides a feedback response to heightened activity within the nervous system, thereby aiding the tuning of neuronal intrinsic plasticity.

In our study, we innovatively identify PTPRN as a novel ubiquitin ligase adaptor in the central nervous system. In response to enhanced network activities, the plastic change of PTPRN facilitates Na_V_1.2 sodium channel endocytosis by recruiting the E3 ligase NEDD4L, comprising a newly discovered regulatory axis (**Figure** **8**). Given the involvement of Na_V_1.2 channels in various neurological and psychiatric brain disorders beyond epilepsy,^[^ ^42^ ^]^ our results suggest that modulating intrinsic neuronal excitability via the PTPRN‐Na_V_1.2 axis could play a significant role in both physiological and pathological processes of the brain.

Neurons in the central nervous system are able to modify their input‐output properties in response to activity, a phenomenon known as intrinsic plasticity.^[^ ^6a^ ^]^ This allows neurons to adapt to changes in their environment during development, learning, and in conditions such as epilepsy.^[^ ^43^ ^]^ Numerous pieces of evidence have shown that the intrinsic plasticity mechanisms enabling DG granule cells to dampen excitation may become apparent during epileptogenesis, with ion channels playing a significant role.^[^ ^44^ ^]^ For instance, epileptic granule cells display strongly enlarged inwardly rectifying currents contributed by rectifier potassium channels (Kir), and this has been observed in both epilepsy patients and the KA mouse model.^[^ ^7a,b^ ^]^ It has also been reported that the combined upregulation of Kir and hyperpolarization‐activated cyclic nucleotide‐gated channels (HCN) conductance attenuates excitatory synaptic input in granule cells of patients with TLE, leading to the stabilization of membrane potential and responsiveness.^[^ ^7c^ ^]^ In our study, we demonstrated that PTPRN expression responds to neuronal activities (Figure 1E–K). Its increase leads to facilitated Na_V_1.2 channel endocytosis and reduced intrinsic excitability (Figures 2, 3, 4, 5, 6). These results suggest that PTPRN could be a mediator linking neuronal activity and intrinsic plasticity, and reveal a mechanism involving ion channel trafficking that allows DG granule cells to homeostatically scale their excitability. While we demonstrated PTPRN reduces intrinsic excitability through its interaction with Na_V_1.2 channels, IP‐MS results suggest PTPRN also interacts with other ion channels including AMPA receptors, NMDA receptors, and Ca_V_ channels (Table S6, Supporting Information), which are also strongly associated with epilepsy.^[^ ^45^ ^]^ However, our results showed that the knockdown of Na_V_1.2 alone was capable of reversing aberrant intrinsic excitability and seizure susceptibility in PTPRN‐KO mice (Figure 7), suggesting that the augmentation of the Na_V_1.2‐mediated current is the major contributor to the phenotype of transgenic mice with PTPRN deficiency.

PTPRN is an evolutionarily conserved member of the RPTP family,^[^ ^13^ ^]^ but it lacks PTP activity.^[^ ^46^ ^]^ Previous studies have shown that the cleaved cytosolic fragment of PTPRN enhances the transcription of secretory granule genes by binding to tyrosine phosphorylated signal transducers and activators of transcription 5 (pSTAT‐5) and preventing its dephosphorylation in pancreatic β‐cells.^[^ ^14^ ^]^ This raises the question of whether PTPRN modulates Na_V_1.2 channels at the transcriptional level or phosphorylation level. However, in our study, we found that neither PTPRN deletion nor overexpression in DG granule cells had any effect on Na_V_1.2 transcription or dephosphorylation (Figure S4, Supporting Information). Moreover, IP‐MS results revealed that PTPRN does not interact with any member of STAT family in the hippocampus (Table S6, Supporting Information). These findings suggest that PTPRN‐mediated Na_V_1.2 modulation occurs exclusively via the regulation of ion channel trafficking at the molecular level.

The interaction between PTPRN and Na_V_1.2 channels leads to a reduction in Na_V_1.2 expression on the cell surface. Our data suggests that PTPRN facilitates the internalization of Na_V_1.2 by recruiting the ubiquitin E3 ligase NEDD4L and promoting NEDD4L‐mediated ubiquitination (Figures 5 and 6). NEDD4L has been reported to bind the PPSY motif in Na_V_ channels and accelerate their endocytosis as well as degradation via ubiquitination.^[^ ^40^, ^47^ ^]^ Interestingly, we found that although PTPRN acts an adaptor to strengthen the interaction between Na_V_1.2 and NEDD4L, it does not affect the total level of Na_V_1.2 protein in the hippocampus (Figure S4, Supporting Information), suggesting that PTPRN induces internalization without promoting degradation. This finding is consistent with one previous report that αB‐crystallin weakens the Na_V_1.5‐NEDD4L complex, leading to enhanced Na_V_1.5 channel surface expression without altering the total protein level.^[^ ^47a^ ^]^ It is worth noting that NEDD4L has been discovered to induce K29‐, K48‐, and K63‐linked ubiquitination.^[^ ^48^ ^]^ Therefore, it is reasonable to speculate that PTPRN plays a role as an adaptor to selectively promote K63‐linked ubiquitination, which primarily affects subcellular localization of ubiquitinated proteins.^[^ ^49^ ^]^

Our findings provide evidence of a protective role of PTPRN in the context of TLE that PTPRN promotes NEDD4L‐mediated endocytosis and thus down‐regulates intrinsic neuronal excitability. Comprehension of this molecular mechanism opens an avenue for selective modulation on Na_V_1.2 channel function, holding promise for the treatment of Na_V_1.2‐related epilepsy in the future.

## 4 Conclusion

In summary, PTPRN is identified as a significant regulator of activity‐dependent intrinsic neuronal plasticity. A notable increase in PTPRN expression has been observed at both the mRNA and protein levels in response to heightened neuronal activity. This increase correlates with a reduction in intrinsic excitability of DG granule cells and a concurrent suppression of seizure susceptibility. Further investigation has led to the elucidation of PTPRN's role as an adaptor, whereby it recruits the ubiquitin E3 ligase NEDD4L to Na_V_1.2 channels. This mechanism facilitates NEDD4L‐mediated ubiquitination and subsequent endocytosis of these channels, providing an exclusive molecular pathway underlying Na_V_1.2 channel modulation.

## 5 Experimental Section

### Animals

Male adult Sprague‐Dawley rats (180–250 g) and C57BL/6 mice (6‐ to 8‐week‐old) were purchased from Charles River Laboratories. PTPRN knockout C57BL/6 mice were generated by Cyagen Biosciences. Na_V_1.6 knockout C3HeB/FeJ mice were generous gifts from Professor Yousheng Shu at Fudan University. Heterozygous mice were intercrossed to obtain homozygous mice. All animals were housed on a 12 h light/dark cycle with ad libitum access to food and water. All experiments using animals were conducted under the Institutional Animal Care and Use Committee‐approved protocols at Peking University in accordance with National Institutes of Health and institutional guidelines. Every effort was made to minimize animal suffering and the number of animals used. The experiments were blind to viral treatment or genotype during behavioral testing.

### Generation of PTPRN Knockout Mice

PTPRN knockout mice were generated by Cyagen Biosciences using the CRISPR‐Cas9‐directed genome editing, as previously described.^[^ ^19^ ^]^ The sequences of the two guide RNAs (gRNAs) were as follows: gRNA #1 (forward strand): AGCTAATGGCACGTCGGGAAAGG; gRNA #2 (reverse strand): GCCTCCCAGGAATATGATTCTGG. gRNAs were transcribed in vitro using the MEGA shortscript T7 kit (Life Technologies). Mouse oocytes were microinjected with a mixture of Cas9 messenger RNA (mRNA) (Invitrogen), gRNA #1, and gRNA #2 and were re‐implanted into C57BL/6 pseudopregnant females. Successful deletions were detected by polymerase chain reaction (PCR) genotyping of mouse tails and confirmed by Sanger sequencing. PTPRN heterozygote pairs were maintained and used for breeding. PCR primers for genotyping were as follows: KO forward: 5′‐CTAGAGAGGCCATTTGCTCGAGT‐3′; KO reverse: 5′‐ACAAGGTTTCTAAGCCACAGGCT‐3′; WT forward: 5′‐AGTGACCCAGTCTTGTGTGGAGT‐3′; WT reverse: 5′‐ACAAGGTTTCTAAGCCACAGGCT‐3′; Amplification of the KO and WT mice resulted in PCR products of 565 and 367 base pairs, respectively.

### Antibodies and Reagents

Commercial antibodies used were: anti‐PTPRN (Abcam, ab207750), anti‐β‐Actin (Biodragon, B1029), anti‐c‐Fos (MilliporeSigma, ABE457), anti‐Na_V_1.2 (Alomone, ASC‐002), anti‐Na_V_1.6 (Alomone, ASC‐009), anti‐HA (Abbkine, A02040), anti‐Flag (Abbkine, A02010), anti‐ Na,K‐ATPase α1 (Abbkine, ABL1141), anti‐Strep (Abbkine, ABT2230), anti‐Myc (Abbkine, ABT2060), anti‐NEDD4L (Proteintech,13690‐1‐AP), anti‐Phospho‐tyrosine (Abbkine, ABM40204), anti‐Ubiquitin (Abbkine, ABP52661), HRP Goat Anti‐Mouse IgG (Biogragon, BF03001), HRP Goat Anti‐Rabbit IgG (Biogragon, BF03008), HRP Mouse Anti‐Rabbit IgG LCS (Abbkine, A25022), Alexa Fluor 488‐AffinityPure Fab Fragment Donkey anti‐rabbit IgG (Jackson, 711‐547‐003). Protein G Dynabeads and EZ‐Link Sulfo‐NHS‐LC‐Biotin were from Pierce. Protease inhibitor mixture cocktail was from Roche Applied Science. Rabbit IgG and mouse IgG were from Santa Cruz. BeyoMag Streptavidin Magnetic Beads, phosphatase inhibitor cocktail and NP40 lysis buffer were from Beyotime. N‐Ethylmaleimide (NEM) and 4′,6‐Diamidino‐2‐phenylindole dihydrochloride (DAPI) were from Solarbio. Tetrodotoxin (TTX) was from Absin Bioscience. Bicuculline, CGP 55 845, and (2R)‐amino‐5‐phosphonovaleric acid (APV) were from Abcam. All other reagents were purchased from Sigma‐Aldrich.

### Weighted Gene Co‐Expression Analysis

R package WGCNA was deployed to construct the weighted co‐expression network to identify modules and hub genes that related to temporal lobe epilepsy. First, gene expression profiles of GSE47752 were downloaded from the gene expression omnibus (GEO) database. Data of kainite acid model and pilocarpine model was analyzed separately. The data was filtered by removing genes with missing values and then used to calculate Pearson correlation between all gene pairs. Next, the *pickSoftThreshold* function in WGCNA was used to determine the proper soft‐thresholding power (β) that fits the criterion of the approximate scale‐free topology of the network, and an adjacency matrix was then built with soft‐thresholding power of 3 (KA) and 15 (Pilocarpine) in this study. The resulting adjacency matrix was used to calculate topological overlap matrix (TO), which was then hierarchically clustered with (1‐TO) as a distance measure. Genes were then assigned into co‐expression modules by dynamic tree cutting algorithm requiring minimal module size of 30 genes. Modules with a distance between the module eigengenes (MEs) of less than 0.25 were merged. Module membership (Pearson correlation between each gene and ME) was then calculated and each gene was assigned to a module for which it had the highest module membership. The Gene list of interesting modules was extracted and a scatterplot of these genes was made. Gene significance was calculated which indicated the Pearson correlation between each gene and phenotype (epilepsy = 1, control = 0)

### Western Blot Analysis

Tissues or cells were homogenized in NP40 lysis buffer on ice and then centrifuged at 14 000 g for 20 min at 4 °C. The supernatant was stored at −80 °C until use. Protein concentration was measured by the BCA method. The lysate was mixed with 5×SDS sample buffer (200 mm Tris‐HCl pH 6.8, 10% SDS, 25% glycerol, 5% 2‐mercaptoethanol, 0.05% bromophenol blue) and denatured for 30 min at 37 °C. Then proteins were loaded on sodium dodecyl sulphate–polyacrylamide gel electrophoresis and transferred onto nitrocellulose filter membrane (PALL). Non‐specific binding sites were blocked with Tris‐buffered saline‐Tween (0.02 m Tris, 0.137 m NaCl and 0.1% Tween 20) containing 5% non‐fat dried milk. Subsequently, proteins of interest were probed with primary antibodies for overnight at 4 °C. After incubation with a secondary antibody, immunoreactive bands were visualized using HRP Substrate Peroxide Solution (Millipore) according to the manufacturer's recommendation. The bands were quantified by densitometry with ImageJ software.

### Real‐Time RT‐PCR

Total RNA was isolated from samples with Trizol reagents (Invitrogen) and used for the first strand cDNA synthesis with AT301‐03 (Transgene), in accordance with the manufacturer's protocol. Relative quantitation was determined using the qPCR Master Mix (Promega) in the MX3005p machine that measures real‐time SYBR green fluorescence and then calculated by means of the comparative Ct method (2^−ΔΔCt^) with the expression of β‐Actin as an internal control. The sequences of the primers used are provided in Table S25 (Supporting Information).

### Immunochemistry

Mice were deeply anesthetized by sodium pentobarbital (SP) and transcardially perfused using 0.01 m phosphate‐buffered saline (PBS, pH 7.4) followed by 4% paraformaldehyde. Brains were post‐fixed and stored in 30% sucrose overnight. Brains were embedded and mounted in paraffin and 20 µm sections were cut using a cryostat (Leica). Sections cut from formalin‐fixed paraffin‐embedded blocks were deparaffinized and rehydrated with serial passage through changes of xylene and graded ethanol. All slides were subjected to heat‐induced epitope retrieval in a citrate buffer kit. Endogenous peroxidase in tissues was blocked by incubation of slides in 3% hydrogen peroxide solution for 25 min prior to incubation with primary antibody. Then slides were incubated with blocking solution (3% bovine serum albumin in phosphate‐buffered saline with Tween 20) for 30 min followed by primary antibody incubation overnight at 4 °C. Antigen‐antibody binding was visualized via application of the EnVision Detection Kit (DAKO). The slices were then washed with PBS followed by counterstaining with haematoxylin. The results of immunohistochemistry experiments were analyzed by Image‐pro plus 6.0.

### Control Tissues or Tissues with TLE

Patients with medically intractable TLE underwent phased presurgical assessment at Shengjing Hospital affiliated to China Medical University. Epilepsy diagnosis (including types and localization) was determined by clinical history, imaging examination (including MRI and/or PET), EEG (including scalp and/or intracranial EEG), and psychological assessment. Patients with TLE caused by stroke, tumor, injury, and malformations were excluded in this study. In those selected for surgery, the temporal lobe was resected according to standard procedures. The study using clinical samples, which include 8 epileptogenic temporal lobe tissues from TLE patients and 8 temporal lobe tissues from non‐epilepsy patients, was approved by the Ethics Committee of Shengjing Hospital affiliated to China Medical University. Tissues were frozen in liquid nitrogen immediately after surgical removal and maintained at −80 °C until protein extraction. Informed consent was obtained from all subjects or their relatives.

### Immunofluorescence

Mice were deeply anesthetized by SP and transcardially perfused using PBS followed by 0.5% paraformaldehyde and 0.5% sucrose (w/v). Brains were post‐fixed and stored in 30% sucrose overnight. Cryostat coronal sections (20 µm) were obtained using a freezing microtome (Leica). The sections were rinsed in PBS and incubated in a blocking solution (5% normal goat serum, 0.3% Triton X‐100 in PBS, vol/vol) at 20–25 °C for 2 h, followed by overnight incubation at 4 °C with primary antibody in blocking solution. The sections were then incubated with second antibody at 20–25 °C for 2 h. The sections were subsequently washed and mounted. Images were taken in the linear range of the photomultiplier with a laser scanning confocal microscope (Zeiss).

### Primary Neuron Isolation and Culture

Primary cortical neurons were isolated from either sex of postnatal homozygous Na_V_1.6 knockout C3HeB/FeJ mice or PTPRN knockout C57BL/6 mice and their WT littermates. After the mice were decapitated, the cortices were removed and separated from the meninges and surrounding tissue. Tissues were digested in 0.25% Trypsin (Lonza) for 30 min followed by centrifugation and resuspension. Subsequently, the cells were plated on poly‐D‐lysine (0.05 mg ml^−1^) pre‐coated glass coverslips in plating medium (DMEM containing 15% FBS) and cultivated at 37 °C and 5% CO_2_ in a humidified incubator. Six hours after plating, the medium was replaced with Neurobasal Plus medium (Invitrogen) containing 2% v/v B‐27 supplement (Invitrogen), 2 mm Glutamax (Invitrogen), 50 U mL^−1^ penicillin and streptomycin (Life Technologies). The primary neurons were grown 7–14 days before experiments with half of the media replaced every three days.

### Cell Culture and Transfection

The human embryonic kidney cells (HEK293 and HEK‐293T) were obtained from ATCC. HEK293 and HEK‐293T cells were maintained in Dulbecco's Modified Eagle Medium (DMEM, Gibco) supplemented with 15% Fetal Bovine Serum (FBS, PAN‐Biotech) at 37 °C and 5% CO_2_. Cells with 70–80% confluence were transiently transfected with plasmid DNA using Lipofectamine 2000 (Invitrogen) according to the manufacturer's instructions. Then the cells were cultured for 20–48 h and subjected to experiments.

### Primary Neuron Pharmacological Treatment and Lentivirus Infection

At 7–14 days in vitro (DIV), cultured primary neurons were treated with 1 µm TTX or 50 mm KCl for 24 h, and then the neurons were collected for RT‐PCR and western blot analysis. For the lentivirus infection, DIV 1–2 primary neurons were prepared for the infection with lentiviruses containing shRNA at MOI (Multiplicity of Infection) = 10. After 8 h of infection, half of the media was replaced. At DIV 10–14, the neurons were subjected to experiments. The infection efficiency was confirmed by the expression of EGFP or cherry under microscopy. Lentiviruses carrying shRNA‐targeting mouse PTPRN and NEDD4L lentiviral vectors (GV493), were from Shanghai Genechem Co., Ltd. The mouse PTPRN#1 shRNA sequence was: 5′‐ CTTATTGACATGGTCCTGAAT −3′; mouse PTPRN#2 shRNA sequence was: 5′‐ GCATACATGGAGGATCACCTT −3′; mouse PTPRN#3 shRNA sequence was: 5′‐ GGCCGAGGAGTATGGCTATAT −3′; mouse NEDD4L shRNA sequence was: 5′‐ CCAGAGAGTTTAAGCAGAAAT −3′; Nonsilencing shRNA sequence was: 5′‐ TTCTCCGAACGTGTCACGT −3′.

### Kainic Acid‐Induced Status Epilepticus

Adult male C57BL/6 mice (6‐ to 8‐week‐old) were injected intraperitoneally with KA to produce seizures with class IV or higher. The dose of KA used was 25 mg kg^−1^ (PTPRN‐KO and PTPRN‐WT) or 28 mg kg^−1^ (PTPRN‐OE and control). To assess epilepsy susceptibility, seizures were evaluated using a modified Racine^[^ ^22^ ^]^: 1) immobility followed by facial clonus; 2) masticatory movements and head nodding; 3) continuous body tremor or wet‐dog shakes; 4) unilateral or bilateral forelimb clonus; 5) rearing and falling. Status epilepticus (SE) was terminated 75 min after onset with the use of SP (30 mg kg^−1^). Mice in the control group were intraperitoneally injected with the same volume of normal saline (NS) and treated with SP (30 mg kg^−1^).

### Pilocarpine‐Induced Status Epilepticus

Adult male Sprague Dawley rats (180–250 g) were injected intraperitoneally with lithium chloride (127 mg kg^−1^) followed by pilocarpine (30 mg kg^−1^) 24 h later.^[^ ^50^ ^]^ Pilocarpine (10 mg kg^−1^) was administered repeatedly every 30 min until the rats developed seizures. SE was terminated with the use of SP (30 mg kg^−1^). Rats in the control group were intraperitoneally injected with the same volume of NS and treated with SP (30 mg kg^−1^).

### EEG Recordings

Adult male C57BL/6 mice (6‐ to 8‐week‐old) were initially anesthetized with 3% isoflurane and secured in the stereotaxic apparatus (RWD Ltd, China). Subdural screw electrodes were secured in the four craniotomies positioned over the left and right dorsal hippocampus and frontal cortex. The subdural screw electrodes were attached to a 6‐pin connector that was centered over the skull and secured with dental cement. All mice were monitored for at least an hour post‐surgery and at 12 h intervals for the next 5 days. EEG and synchronized video were recorded for 12 h a day with BIOPAC MP150 data acquisition system (BIOPAC, USA) and an infrared camera, respectively. EEG signals were acquired at 500 Hz and bandpass filtered at 1–100 Hz. All EEG recordings were analyzed offline using Sirenia Seizure Pro (v. 1.8.4, Pinnacle Technology). Seizures were defined as rapid and rhythmic (>3 Hz) deflections in all EEG channels that lasted >10 s and were at least 3 standard deviations above the baseline root mean square (RMS) amplitude.^[^ ^51^ ^]^ Seizures were considered convulsive if the video record showed behaviors consistent with stages 3–5 on the Racine scale.^[^ ^51^, ^52^ ^]^

### Acute Slice Preparation and Current‐Clamp Recordings

Horizontal slices containing hippocampus were obtained from adult male C57BL/6 mice (6‐ to 8‐week‐old). All data were acquired and analyzed blind to viral treatment or genotype. In brief, mice were anesthetized and perfused intracardially with ice‐cold modified “cutting solution” containing (in mm): 110 choline chloride, 2.5 KCl, 0.5 CaCl_2_, 7 MgCl_2_, 25 NaHCO_3_, 1.25 NaH_2_PO_4_, 10 glucose; bubbled continuously with 95%O_2_/5%CO_2_ to maintain PH at 7.2. The brain was then removed and submerged in ice‐cold “cutting solution”. Next, the brain was cut into 300 µm slices with a vibratome (Leica). Slices were incubated in oxygenated (95% O_2_ and 5% CO_2_) “recording solution” containing (in mm): 125 NaCl, 2.5 KCl, 2 CaCl_2_, 2 MgCl_2_, 25 NaHCO_3_, 1.25 NaH_2_PO_4_, 10 glucose (315 mOsm, PH 7.4, 37 °C) for 20 min, and stored at room temperature.

Slices were subsequently transferred to a submerged chamber containing “recording solution” maintained at 34–36 °C. Whole‐cell recordings were obtained from hippocampal DG granule cells under a ×60 water‐immersion objective of an Olympus BX51WI microscope (Olympus). Pipettes had resistances of 5–8 MΩ. Electrophysiological recordings were made using a Multiclamp 700B amplifier (Molecular Devices). Recordings were filtered at 10 kHz and sampled at 50 kHz. Data were acquired and analyzed using pClamp 10.6 (Molecular Devices). Series resistance was in the order of 10–30 MΩ and was ≈60–80% compensated. Recordings were discarded if the series resistance increased by more than 20% during the course of the recordings.

For current‐clamp recordings, the external solution was supplemented with (in mm) 0.05 APV, 0.01 6‐cyano‐7‐nitro‐quinoxaline‐2,3‐dione, 0.01 bicuculline, and 0.001 CGP 55 845, and internal pipette solution containing (in mm): 118 KMeSO_4_, 15 KCl, 10 HEPES, 2 MgCl_2_, 0.2 EGTA, and 4 Na_2_ATP, 0.3 Tris‐GTP, 14 Tris‐phosphocreatinin (290–300 mOsm, pH 7.3 with KOH). Hippocampal DG granule cells were patched at a holding potential of −80 mV. A series of current pulse was applied to the recording neurons (from −200 to 300 pA, 400 ms duration, 50 pA increments). AP threshold and peak dV/dt measurements were determined from the first AP evoked by 300 pA current. AP indicators were analyzed using Clampfit 10.7 (Molecular Devices). Threshold was defined as the Vm when dV/dt measurements first exceeded 15 V s^−1^.^[^ ^27^ ^]^

### Immunoprecipitation

The hippocampal tissues or HEK‐293T cells co‐expressing full‐length or fragments of PTPRN and Na_V_1.2 were homogenized and lysed in NP40 lysis with cocktail for 30 min at 4 °C. For the determination of ubiquitination, 10 mm NEM was added to prevent deubiquitylation.^[^ ^53^ ^]^ For the determination of tyrosine phosphorylation, phosphatase inhibitor cocktail was added to prevent dephosphorylation. The homogenate was centrifuged for 20 min at 15 000 g and 4 °C to remove cell debris and then supernatant was incubated with antibody for 12 h at 4 °C with constant rotation. Protein G Dynabeads was then added and the rotation was continued until the next day. Beads were then washed three times with NP40 lysis. Between washes, the beads were collected by DynaMag. The remaining proteins were eluted from the beads by re‐suspending the beads in 1×SDS‐PAGE loading buffer and incubating for 30 min at 37 °C. The resultant materials were then subjected to western blot analysis or mass spectrometry.

### Mass Spectrometry

Affinity‐purified proteins were separated by SDS‐PAGE and visualized with Coomassie G‐250. After reduction and alkylation of cysteine residues using dithiothreitol and iodoacetamide, gel pieces were washed, dehydrated, and subsequently swollen with ammonium bicarbonate buffer containing 10 ng µl^−1^ trypsin (Promega). After an overnight digestion at 37 °C, the reaction was quenched by the addition of trifluoroacetic acid (TFA). The tryptic peptides were resuspended and separated by C18 reversed‐phase HPLC (RP‐HPLC) (Thermo Fisher Scientific). The fractions were collected, dried, and then resuspended in TFA. Each fraction was further separated on an analytical C18 column. The peptides were identified by orbitrap mass spectrometry (Thermo Fisher Scientific). Proteins with high credibility (Unique Peptides ≥ 4, Abundance Ratio ≥ 4) were listed in Table S6 (Supporting Information). These proteins were subjected to GO enrichment analysis and KEGG enrichment analysis. The mass spectrometry proteomics data were deposited to the ProteomeXchange Consortium (http://proteomecentral.proteomexchange.org) via the iProX partner repository with the dataset identifier PXD043822.

### Adeno‐Associated Virus Construction and Injection

Adeno‐associated viruses (AAVs) carrying shRNA targeting mouse Scn2a and AAVs carrying PTPRN (NM_0 08985) were from Shanghai GeneChem. Mouse Scn2a shRNA sequence was: 5′‐ ATCAAATCCCTCCGAACATTA −3′. AAV serotype 9 was used in the experiment. For viral injection, 6‐ to 8‐week‐old C57BL/6 mice were anesthetized with isoflurane and placed in a stereotaxic apparatus (RWD Life Science). AAV was bilaterally injected into dorsal hippocampus DG area (coordinates, bregma: anterior‐posterior, −1.60 mm; dorsal‐ventral, −2.00 mm; lateral, ±1.30 mm). Ten minutes after microinjection, the needle was retracted and after another 10 min, the wound was sutured. The tested mice were used at 14 days post injection. All the mice were sacrificed after experiments to confirm the injection sites and the viral trans‐infection effects by checking EGFP under a fluorescence microscope (Zeiss).

### Voltage‐Clamp Recordings

The pB‐2A plasmids expressing full‐length or fragments of PTPRN and Na_V_ channels (excluding full‐length Na_V_1.6) were co‐transfected into HEK‐293T cells using lipofectamine 2000 (Introvigen). To co‐express PTPRN with Na_V_1.6, the plasmid expressing PTPRN was transfected into a stable HEK293 cell line expressing Na_V_1.6. 20–28 h after transfection, whole‐cell recordings were performed.

Sodium currents were recorded at room temperature using an EPC‐10 amplifier (HEKA Electronik) at a 20 kHz sample rate and were low‐pass filtered at 5 kHz. The patch pipettes were pulled from borosilicate glass capillaries with a micropipette puller (Narashige PC‐10) and fire‐polished using a microforge (Narashige MF‐830) to a resistance of 1.8–2.5 MΩ. The series resistance was controlled under 5 MΩ and was compensated above 70%. The data were acquired by the PatchMaster program (HEKA Electronik). The extracellular recording solution contained (in mm): 140 NaCl, 3 KCl, 1 CaCl_2_, 1 MgCl_2_, 10 Glucose, and 10 HEPES (310 mOsm L^−1^, pH 7.30 with NaOH). The recording pipette intracellular solution contained (in mm): 140 CsF, 10 NaCl, 1 EGTA, and 10 HEPES (300 mOsm L^−1^, pH 7.30 with CsOH).

In whole‐cell recordings of Na_V_1.2 or Na_V_1.6 currents, the cells were held at −90 mV and the current‐voltage (*I–V*) relationships were obtained by a 50 ms step from −90 to 45 mV in 5 mV increment. The current density was assessed by the currents against capacitance. To calculate the voltage dependence of activation, conductance *G* was fitted with the Boltzmann function *G*/*G_max_* = (1 + exp [(*V – V*_1/2_) / *k*])^−1^, where *V*_1/2_ indicates the voltage at half‐maximal activation, and *k* was a slope factor describing voltage sensitivity of the channel. *G* were calculated from the *I–V* relationships according to *G *= *I* / (*V*‐*E*), where *I* was the peak current measured at test potential *V*, and *E* was the calculated reversal potential. *G_max_* was the maximum conductance between −90 and 20 mV. Voltage dependence of steady‐state inactivation was assessed by currents generated by the −5 mV postpulse voltage step normalized to the maximum current against prepulse voltage step from −100 to 35 mV in 5 mV increment. The normalized current was fitted with the Boltzmann function. Time‐dependent recovery from inactivation was evaluated by fitting peak current recovery with a two‐exponential function. The peak current was elicited at −5 mV in the measurement of time‐dependent recovery. Specifically, in whole‐cell recordings of Na_V_1.5 currents, the cells were held at −120 mV and the current‐voltage (*I–V*) relationships were obtained by a 50 ms step from −100 to 35 mV in 5 mV increment. Voltage dependence of steady‐state inactivation was assessed by currents generated by the −20 mV postpulse voltage step normalized to the maximum current against prepulse voltage step from −120 to 15 mV in 5 mV increment. Also, peak current was elicited at −20 mV in the measurement of time‐dependent recovery.

For the whole‐cell recording of the primary cortical neurons, the pipette resistance was 2.8–3.8 MΩ in the whole‐cell recording of primary neurons and series resistance was controlled under 10 MΩ and was compensated above 60%. Neurons with short dendrites were preferred to minimize the space clamp errors. The extracellular recording solution contained (in mm): 140 NaCl, 3 KCl, 1 CaCl_2_, 1 MgCl_2_, 10 Glucose, 10 HEPES, 30 TEACl, 0.5 CdCl (310 mOsm L^−1^, pH 7.30 with NaOH). The recording pipette intracellular solution was the same as described above. To obtain purified sodium current, 500 nm TTX was applied and sodium current was measured as the TTX‐sensitive inward currents using the same protocol for *I–V* relationship of Na_V_1.2. The stock solutions of TTX were prepared by dissolving TTX at a concentration of 500 µm in citrate^−^/Na^+^ buffer (10 mmol L^−1^).

### Surface Biotinylation

Primary neurons and HEK‐293T cells expressing Na_V_1.2 plus PTPRN or CD4 as control were incubated with 1 mg ml^−1^ of EZ‐Link Sulfo‐NHS‐SS‐Biotin in cold PBS for 1 h at 4 °C, constantly moving. Free biotin was quenched, twice, with 100 mm Tris in cold PBS, and once with cold PBS to remove biotin excess. The cells were then harvested with NP40 lysis and centrifuged at 15 000 g at 4 °C for 20 min. The supernatant was incubated with BeyoMag Streptavidin Magnetic Beads, and the remaining supernatant was kept as input. The beads were subsequently washed with NP40 lysis buffer. The remaining proteins were eluted from the beads by re‐suspending the beads in 1×SDS‐PAGE loading buffer and incubating for 30 min at 37 °C. The resultant materials were then subjected to western blot analysis

### Trafficking Inhibition

To investigate the mechanism of the regulation on Na_V_1.2 by PTPRN, 80 µm Dynasore (Abcam),^[^ ^54^ ^]^ 1 µm TAK‐243 (TargetMol),^[^ ^35^ ^]^ 20 µm Pitstop2 (GLPBIO),^[^ ^55^ ^]^ or 50 µm Heclin (GLPBIO)^[^ ^48c^ ^]^ were added into the medium 2 h before the electrophysiology recording. Besides, 2 µg ml^−1^ Brefeldin A (Solarbio) was applied into the medium 1 h before the electrophysiology recording.^[^ ^56^ ^]^ Specifically, the medium was replaced by the Opti‐MEM (Gibco) when the Dynasore was applied to avoid the impact of the Dynasore's activity from the serum.^[^ ^30^ ^]^

### Peptide Purification

Twin‐Strep‐tag were tandemly inserted into the C‐terminal of PTPRN intracellular fragment (aa 601–979) and TagRFP, respectively. HEK‐293T cells were transfected with plasmids using Lipofectamine 2000 and cultured for 48 h before harvest. Cells were lysed in NP40 lysis with a cocktail for 30 min at 4 °C. The homogenate was centrifuged for 20 min at 15 000 g and 4 °C to remove cell debris. The supernatant was diluted with PBS and loaded into Streptactin Beads 4FF (Smart‐Lifesciences). The purified protein was eluted using elution buffer (5 mm d‐Desthiobiotin (Smart‐Lifesciences) in PBS) and concentrated using a 10 kDa MWCO Amicon (Millipore).

### Statistical Analysis

For in vitro experiments, the cells were evenly suspended and then randomly distributed in each well‐tested. For in vivo experiments, the animals were distributed into various treatment groups randomly. Statistical analyses were performed using GraphPad Prism 9 (GraphPad Software) and SPSS 26.0 software (SPSS Inc.). Before statistical analysis, variation within each group of data and the assumptions of the tests were checked. Comparisons between two independent groups were made using unpaired Student's two‐tailed t test. Comparisons among three or more groups were made using one‐ or two‐way analysis of variance followed by Bonferroni's post hoc test. No statistical methods were used to predetermine sample sizes but the sample sizes were similar to those reported previously in the field.^[^ ^6c^ ^]^ All experiments and analysis of data were performed in a blinded manner by investigators who were unaware of the genotype or manipulation. * *p* < 0.05, ** *p* < 0.01, *** *p* < 0.001, **** *p* < 0.0001. All data were presented as mean ± SEM.

## Conflict of Interest

The authors declare no conflict of interest.

## Author Contributions

Y.W., H.Y., N.L., and L.W. contributed equally to this work. Y.W., L.W., and S.L. performed and analyzed western blotting, RT‐PCR, immunostaining, immunoprecipitation. H.Y. performed and analyzed voltage‐clamp recordings. N.L. performed and analyzed the current‐clamp recordings. N.L. and X.M. performed virus microinjections. Y.W., N.L., L.W., C.G., and H.C. performed kainic acid/pilocarpine‐induced status epilepticus models. X.T., C.G., N.L., L.W., and J.C. performed EEG recordings. W.M. provided human epileptic tissues and helped with western blotting experiments. Y.W., N.L., L.W., H.S., H.C., C.G., J.C., and X.T. identified the transgenic mice. J.Y., J.L., L.W., and Y.W. isolated primary neurons. Y.W., C.P., and W.K. performed bioinformatics analysis. Y.W., H.Y., L.W., S.L., and J.D. performed the molecular cloning. Z.H., X.T., Y.W., H.Y., and N.L. designed the experiments. Z.H. and Y.W. wrote the manuscript. Z.H., X.T., and M.S. reviewed the manuscript. All authors have read and approved the final version of this manuscript and agree to be accountable for all aspects of the work in ensuring that questions related to the accuracy or integrity of any part of the work are appropriately investigated and resolved. All persons designated as authors qualify for authorship, and all those who qualify for authorship are listed.

## Supporting information

Supporting Information

Supplemental Video 1

## Data Availability

The data that support the findings of this study are available in the supplementary material of this article.
