# Supplementary material for: A Novel Ubiquitin Ligase Adaptor PTPRN Suppresses Seizure Susceptibility through Endocytosis of NaV1.2 Sodium Channels
Source: Adv Sci (Weinh). 2024 Jun 14;11(29):2400560. doi: 10.1002/advs.202400560 (PMC11304301; doi:10.1002/advs.202400560)
Supplement: Supplementary file 1 — Supporting Information [file ADVS-11-2400560-s001.docx]

Supporting Information

**A Novel Ubiquitin Ligase Adaptor PTPRN Suppresses Seizure Susceptibility through Endocytosis of Na_V_1.2 Sodium Channels**

*Yifan Wang, Hui Yang, Na Li, Lili Wang, Chang Guo, Weining Ma, Shiqi Liu, Chao Peng, Jiexin Chen, Huifang Song, Hedan Chen, Xinyue Ma, Jingyun Yi, Jingjing Lian, Weikaixin Kong, Jie Dong, Xinyu Tu, Mala Shah, Xin Tian* ＆ Zhuo Huang**

Figure S1-4

Table S1-25


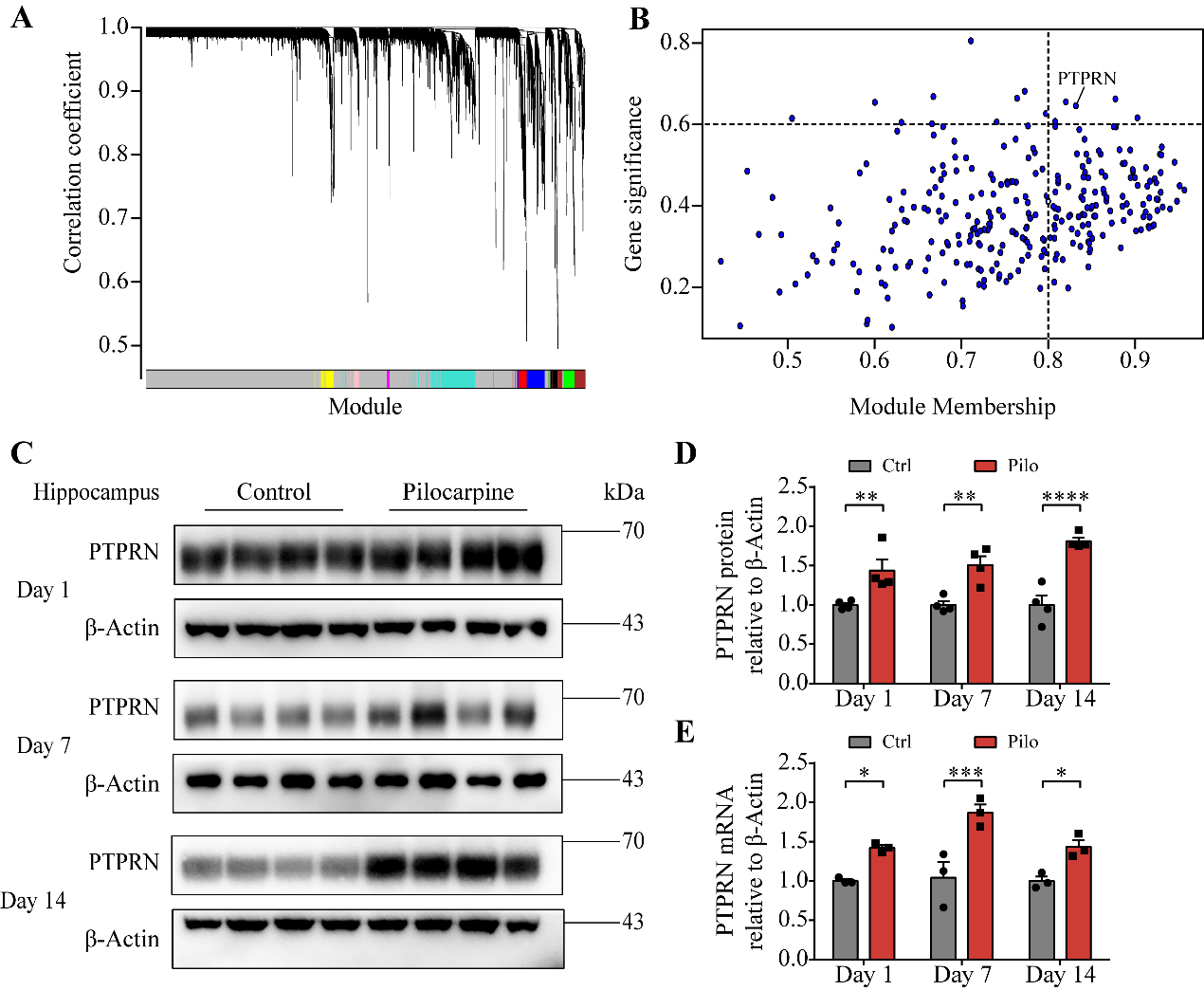


**Figure S1. PTPRN expression levels are high in the rat pilocarpine (pilo) model.** (**A**) Identification of modules in the Pilo model. A total of 9 modules were identified in a hierarchical clustering tree diagram. (**B**) Scatterplot of gene significance versus module membership for genes in the most relevant module. Candidate critical genes were selected according to thresholds: gene significance > 0.6 and module membership > 0.8 (dotted line). (**C**) Western blot analysis of PTPRN in hippocampal tissues obtained from rats injected with Pilo or saline (Ctrl) respectively at 1, 7 and 14 days after injection. (**D**) Quantification of the results in (**C**) by normalizing the protein levels of PTPRN to those of β-Actin. n = 4 mice in each group, ***p* < 0.01, *****p* < 0.0001, two-way ANOVA with Bonferroni’s multiple-comparisons test. (**E**) RT-PCR analysis of PTPRN in hippocampal tissues obtained from mice described in (**C**). The levels of mRNA were normalized to those of β-Actin. n = 4 mice in each group, **p* < 0.05, ****p* < 0.001, two-way ANOVA with Bonferroni’s multiple-comparisons test. Data are represented as mean ± s.e.m.


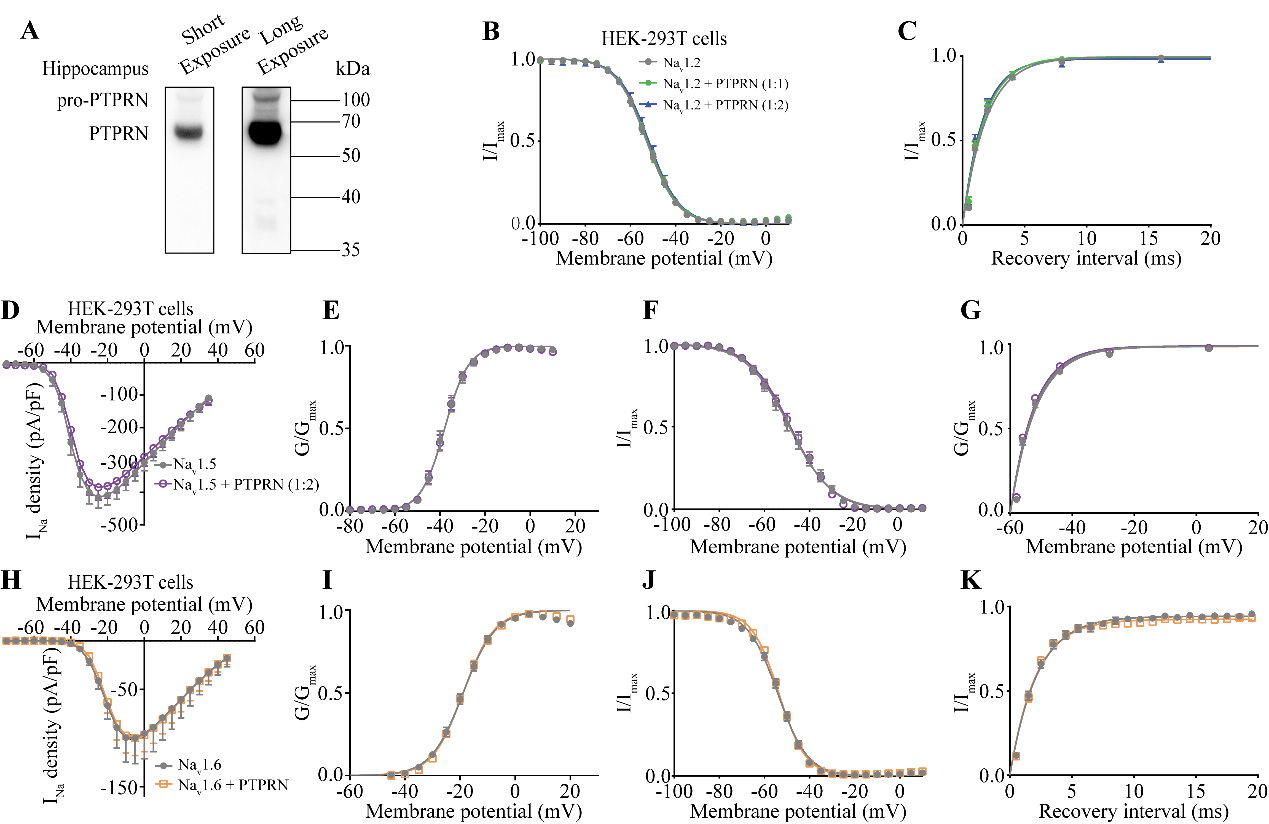


**Figure S2.** **PTPRN selectively modulates the Na_V_1.2-mediated current.** (**A**) Western blot analysis showing PTPRN and pro-PTPRN in mouse hippocampal lysate. (**B**) Graph depicting voltage-dependence of inactivation for Na_V_1.2 channels co-expressed with empty vector (n = 18) or PTPRN in a 1:1 (n =18) or 1:2 (n = 20) ratio. The lines are the best-fitted Boltzmann curves. (**C**) Graph depicting time-dependent rate of recovery from inactivation for Na_V_1.2 channels co-expressed with empty vector or PTPRN. The plateau values were estimated from one-phase decay fit to the data. (**D**) Current density versus voltage relationship for Na_V_1.5 channels co-expressed with empty vector (n = 19) or PTPRN (n = 19). Currents in all figures were normalized to cell capacitance. (**E**) Graph depicting voltage dependence of activation for Na_V_1.5 channels co-expressed with empty vector or PTPRN. The lines are the best-fitted Boltzmann curves. (**F**) Graph depicting voltage dependence of inactivation for Na_V_1.5 channels co-expressed with empty vector or PTPRN. The lines are the best-fitted Boltzmann curves. (**G**) Graph depicting time-dependent rate of recovery from inactivation for Na_V_1.5 channels co-expressed with empty vector or PTPRN. The plateau values were estimated from one-phase decay fit to the data. (**H**) Current density versus voltage relationship for Na_V_1.6 channels co-expressed with empty vector (n = 21) or PTPRN (n = 17). Currents in all figures were normalized to cell capacitance. (**I**) Graph depicting voltage dependence of activation for Na_V_1.5 channels co-expressed with empty vector or PTPRN. The lines are the best-fitted Boltzmann curves. (**J**) Graph depicting voltage dependence of inactivation for Na_V_1.6 channels co-expressed with empty vector or PTPRN. The lines are the best-fitted Boltzmann curves. (**K**) Graph depicting time-dependent rate of recovery from inactivation for Na_V_1.6 channels co-expressed with empty vector or PTPRN. The plateau values were estimated from one-phase decay fit to the data. Data are represented as mean ± s.e.m.


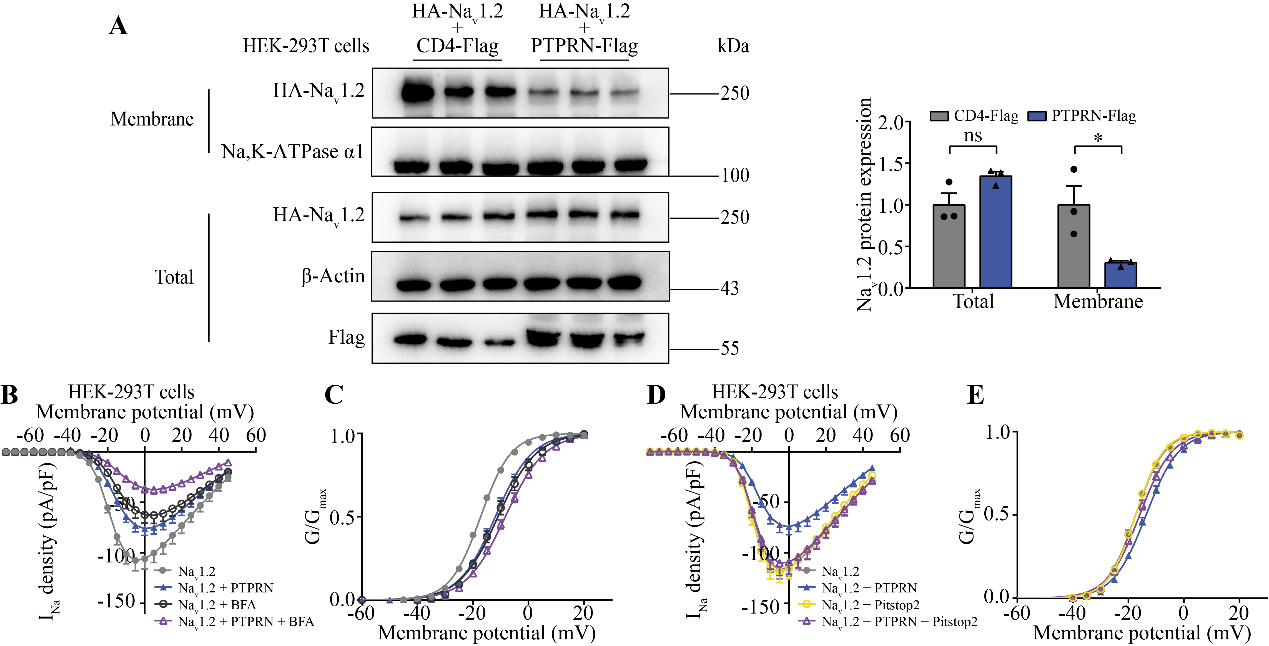


**Figure S3.** **PTPRN promotes Na_V_1.2 channel internalization through clathrin-mediated endocytosis (CME).** (**A**) Left panel: Immunoblot analysis of cell surface biotinylation performed in HEK-293T cells expressing Na_V_1.2 and PTPRN in a 1:2 ratio (Na_V_1.2 + PTPRN) or Na_V_1.2 channels and CD4 as a control (Na_V_1.2 + CD4). Total lysates (total) and biotinylated fractions (membrane) were analyzed by western blot. Right panel: Quantification of Na_V_1.2 total expression and surface expression. n = 3, **p* < 0.05, two-way ANOVA with Bonferroni’s multiple-comparisons test. (**B**) Current density versus voltage relationship for HEK-293T cells expressing Na_V_1.2 and PTPRN in a 1:2 ratio (Na_V_1.2 + PTPRN) or Na_V_1.2 channels and empty vector in a 1:2 ratio (Na_V_1.2), with or without treatment with BFA (2 μg/ml, 1 h). n = 18 for Na_V_1.2 + PTPRN plus BFA, n = 26 for Na_V_1.2 plus BFA, n = 20 for Na_V_1.2 + PTPRN without BFA, and n = 21 for Na_V_1.2 without BFA. (**C**) Graph depicting voltage dependence of activation for Na_V_1.2 channels described in (**B**). The lines are the best-fitted Boltzmann curves. (**D**) Current density versus voltage relationship for HEK-293T cells expressing Na_V_1.2 and PTPRN in a 1:2 ratio (Na_V_1.2 + PTPRN) or Na_V_1.2 and empty vector in a 1:2 ratio (Na_V_1.2), with or without treatment with Pitstop2 (20 μM, 2 h). n = 18 for Na_V_1.2 + PTPRN plus Pitstop2, n = 26 for Na_V_1.2 plus Pitstop 2, n = 26 for Na_V_1.2 + PTPRN without Pitstop 2, and n = 24 for Na_V_1.2 without Pitstop2. (**E**) Graph depicting voltage dependence of activation for Na_V_1.2 channels described in (**D**). The lines are the best-fitted Boltzmann curves. Data are represented as mean ± s.e.m.


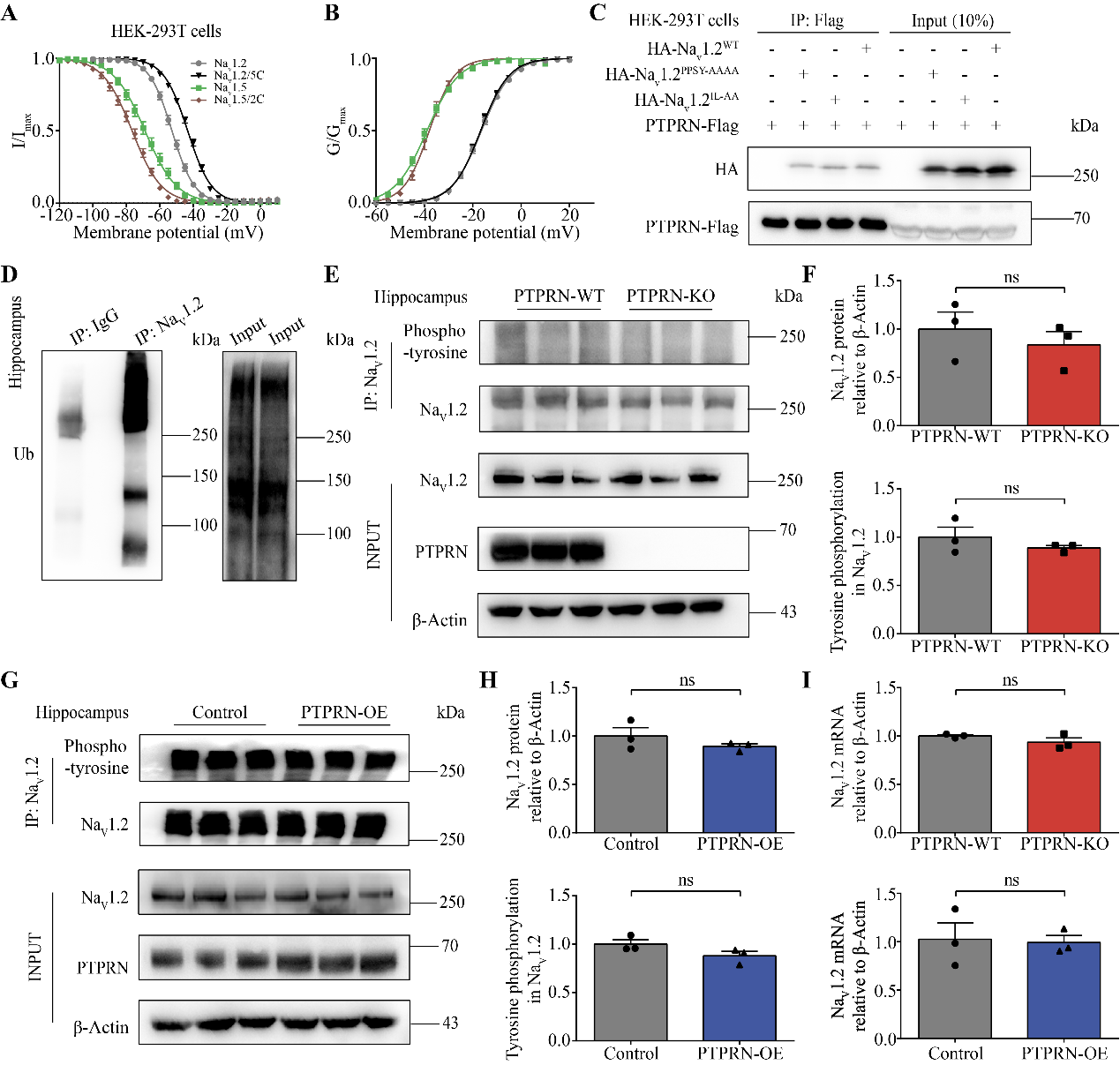


**Figure S4.** **PTPRN facilitates the ubiquitination of Na_V_1.2 channels by recruiting NEDD4L.** (**A**) Graph depicting voltage dependence of inactivation for Na_V_1.2 (n = 21), Na_V_1.5 (n = 19), Na_V_1.2/5C (n = 19), or Na_V_1.5/2C (n = 20) channels expressed in HEK-293T cells. (**B**) Graph depicting time-dependent rate of recovery from inactivation for Na_V_1.2, Na_V_1.5, Na_V_1.2/5C, or Na_V_1.5/2C channels expressed in HEK-293T cells. The plateau values were estimated from one-phase decay fit to the data. (**C**) Immunoblot analysis of HA-tagged WT/mutated Na_V_1.2 and PTPRN-Flag in IP-Flag samples prepared from HEK-293T cell lysate. (**D**) Representative western blot showing Na_V_1.2 ubiquitination in IP-Na_V_1.2 samples prepared from mouse hippocampus lysate. (**E**) Western blot analysis of indicated proteins in IP-Na_V_1.2 samples prepared from hippocampal tissues of PTPRN-KO or PTPRN-WT mice. (**F**) Quantification of Na_V_1.2 protein levels (top) and tyrosine phosphorylation in Na_V_1.2 (bottom) in (**E**). n = 3, unpaired two-tailed Student’s *t*-test. (**G**) Western blot analysis of indicated proteins in IP-Na_V_1.2 samples prepared from hippocampi of mice infected with control AAV or AAV expressing PTPRN. (**H**) Quantification of Na_V_1.2 protein levels (top) and tyrosine phosphorylation in Na_V_1.2 (bottom). n = 3, unpaired two-tailed Student’s *t*-test. (**I**) Top: RT-PCR analysis of PTPRN in hippocampal tissues obtained from PTPRN-KO/WT mice. The levels of mRNA were normalized to those of β-Actin. n = 3, unpaired two-tailed Student’s *t*-test. Bottom: RT-PCR analysis of PTPRN in hippocampal tissues obtained from mice infected with control AAV or AAV expressing PTPRN. The levels of mRNA were normalized to those of β-Actin. n = 3, unpaired two-tailed Student’s *t*-test. Data are represented as mean ± s.e.m.

**Table S1. Module-phenotype relationship of kainic acid model.**

| Module | Pearson Correlation coefficient | *p* value |
| --- | --- | --- |
| Brown | -0.32 | 0.1 |
| Black | -0.27 | 0.2 |
| Midnightblue | 0.13 | 0.6 |
| Darkgreen | 0.087 | 0.7 |
| Tan | 0.15 | 0.5 |
| Greenyellow | 0.16 | 0.5 |
| Darkgrey | 0.38 | 0.07 |
| Lightcyan | 0.4 | 0.05 |
| Yellow | 0.17 | 0.4 |
| Green | 0.17 | 0.4 |
| Salmon | 0.32 | 0.1 |
| Lightgreen | 0.13 | 0.6 |
| Purple | 0.085 | 0.7 |
| Pink | 0.17 | 0.4 |
| Cyan | 0.37 | 0.07 |
| Red | 0.47 | 0.02 |
| Turquoise | 0.37 | 0.07 |
| Grey60 | -0.32 | 0.1 |
| Darkred | 0.14 | 0.5 |
| Lightyellow | 0.18 | 0.4 |
| Magenta | -0.27 | 0.2 |
| Royalblue | 0.076 | 0.7 |
| Blue | 0.092 | 0.7 |
| Darkturquoise | -0.38 | 0.07 |
| Grey | 0.13 | 0.5 |

**Table S2. Module-phenotype relationship of pilocarpine model**

| Module | Pearson Correlation coefficient | *p* value |
| --- | --- | --- |
| Blue | 0.5 | 0.0001 |
| Red | 0.34 | 0.01 |
| Black | 0.11 | 0.4 |
| Brown | 0.47 | 0.0004 |
| Green | 0.009 | 0.9 |
| Pink | 0.095 | 0.5 |
| Yellow | 0.085 | 0.5 |
| Magenta | 0.029 | 0.8 |
| Turquoise | -0.38 | 0.005 |
| Grey | 0.038 | 0.8 |

**Table S3. Demographic and clinical characteristics of selected patients with mesial TLE**

| Patient | Age | Sex | Duration of disease (Years) | Medication history | MRI examination | Type of surgery |
| --- | --- | --- | --- | --- | --- | --- |
| NEP1 | 20 | Male | \ | \ | \ | Temporal lobe Resection |
| NEP2 | 30 | Male | \ | \ | \ |  |
| NEP3 | 22 | Male | \ | \ | \ |  |
| NEP4 | 19 | Male | \ | \ | \ |  |
| NEP5 | 35 | Female | \ | \ | \ |  |
| NEP6 | 17 | Male | \ | \ | \ |  |
| NEP7 | 68 | Male | \ | \ | \ |  |
| NEP8 | 64 | Male | \ | \ | \ |  |
| EP1 | 36 | Female | 14 | Carbamazepine, Valproate | R hippocampus atrophy, L and R hippocampus hypometabolism |  |
| EP2 | 35 | Male | 16 | Levetiracetam, Valproate | R hippocampus sclerosis |  |
| EP3 | 25 | Male | 23 | Topiramate, Carbamazepine, Valproate, Oxcarbazepine, Levetiracetam, Lamotrigine | Multiple hypometabolism |  |
| EP4 | 64 | Female | 30 | Valproate | R temporal lobe abnormal signal |  |
| EP5 | 35 | Female | 27 | Carbamazepine, Oxcarbazepine,  Clonazepam | L and R temporal lobe abnormal signal |  |
| EP6 | 28 | Female | 27 | Oxcarbazepine | L temporal lobe atrophy |  |
| EP7 | 26 | Female | 15 | Oxcarbazepine,  Levetiracetam | R hippocampus atrophy |  |
| EP8 | 32 | Female | 19 | Levetiracetam,  Valproate | R hippocampus sclerosis |  |

*NEP, non-epilepsy patient; EP, epilepsy patient.

**Table S4. Electrophysiological properties of hippocampal DG granule cells of mice**

| Parameters | PTPRN-WT (n=14) | PTPRN-KO (n=14) |
| --- | --- | --- |
| Input resistance (MΩ) | 178.5 ± 10.48 | 184.9 ± 15.82 |
| Threshold (mV) | -46.88 ± 0.5373 | -46.93 ± 0.5964 |
| Peak dV/dt (mV/ms) | 187.6 ± 4.682 | 210.7 ± 6.004** |
| Amplitude (mV) | 120.0 ± 0.8774 | 122.3 ± 0.7461 |
| Half width (ms) | 1.496 ± 0.0334 | 1.512 ± 0.0273 |
| Rise slope (mV/ms) | 137.9 ± 3.827 | 162.6 ± 4.930*** |
| Decay slope (mV/ms) | -42.74 ± 1.179 | -43.51 ± 1.014 |

Data are reported as mean ± s.e.m. for DG granule cells of mice. ***p* < 0.01, unpaired two-tailed Student’s t-test.

**Table S5. Electrophysiological properties of hippocampal DG granule cells of mice**

| Parameters | Control (n=18) | PTPRN-OE (n=21) |
| --- | --- | --- |
| Input resistance (MΩ) | 205.1 ± 11.60 | 203.0 ± 12.69 |
| Threshold (mV) | -49.31 ± 0.6021 | -47.97 ± 0.6057 |
| Rise max (mV/ms) | 199.1 ± 7.247 | 176.5 ± 5.560* |
| Amplitude (mV) | 121.4 ± 0.5809 | 122.4 ± 0.7166 |
| Half width (ms) | 1.528 ± 0.0625 | 1.798 ± 0.0664** |
| Rise slope (mV/ms) | 146.6 ± 7.474 | 130.2 ± 5.051 |
| Decay slope (mV/ms) | -43.38 ± 1.522 | -36.54 ± 1.301** |

Data are reported as mean ± s.e.m. for DG granule cells of mice. * *p* < 0.05, unpaired two-tailed Student’s t-test.

**Table S6. Protein interactors of PTPRN in mouse hippocampus**

| Accession | Gene Symbol | Unique Peptides | Abundance Ratio |
| --- | --- | --- | --- |
| A2AGT5 | CKAP5 | 5 | 4.174 |
| A2AJI0 | MAP7D1 | 4 | 7.308 |
| B0V2N1 | PTPRS | 12 | 31.432 |
| B1AWN6 | SCN2A | 4 | 100 |
| B9EJA2 | CTTNBP2 | 10 | 31.476 |
| D3YWQ0 | DGKI | 6 | 100 |
| D3YZU1 | SHANK1 | 5 | 100 |
| E9PVA8 | GCN1 | 4 | 4.188 |
| E9Q3L2 | PI4KA | 4 | 100 |
| E9Q401 | RYR2 | 8 | 4.577 |
| F6SEU4 | SYNGAP1 | 10 | 28.712 |
| G5E829 | ATP2B1 | 8 | 4.164 |
| G5E8K5 | ANK3 | 13 | 14.793 |
| O08532 | CACNA2D1 | 8 | 8.57 |
| O08539 | BIN1 | 5 | 12.374 |
| O08553 | DPYSL2 | 13 | 7.038 |
| O08599 | STXBP1 | 9 | 8.034 |
| O08638 | MYH11 | 12 | 5.258 |
| O15020 | SPTBN2 | 57 | 84.359 |
| O35737 | HNRNPH1 | 4 | 22.984 |
| O35927 | CTNND2 | 11 | 13.5 |
| O43581 | SYT7 | 5 | 100 |
| O54983 | CRYM | 5 | 4.333 |
| O54991 | CNTNAP1 | 7 | 6.883 |
| O55022 | PGRMC1 | 4 | 8.72 |
| O55042 | SNCA | 9 | 5.401 |
| O55091 | IMPACT | 4 | 7.624 |
| O55106 | STRN | 4 | 9.688 |
| O55131 | SEPTIN7 | 6 | 8.385 |
| O55143 | ATP2A2 | 16 | 10.798 |
| O70161 | PIP5K1C | 7 | 22.494 |
| O88737 | BSN | 23 | 4.607 |
| O88935 | SYN1 | 17 | 8.693 |
| O89051 | ITM2B | 5 | 93.681 |
| P02788 | LTF | 23 | 4.133 |
| P04370 | MBP | 7 | 24.229 |
| P04919 | SLC4A1 | 5 | 9.319 |
| P05063 | ALDOC | 5 | 7.343 |
| P05064 | ALDOA | 11 | 4.181 |
| P05201 | GOT1 | 6 | 8.516 |
| P05202 | GOT2 | 7 | 10.338 |
| P05213 | TUBA1B | 5 | 17.983 |
| P06837 | GAP43 | 6 | 5.299 |
| P07901 | HSP90AA1 | 10 | 8.298 |
| P08032 | SPTA1 | 18 | 14.376 |
| P08238 | HSP90AB1 | 14 | 8.366 |
| P08551 | NEFL | 5 | 100 |
| P0DP27 | CALM2 | 5 | 22.724 |
| P10637 | MAPT | 4 | 7.413 |
| P11142 | HSPA8 | 18 | 11.043 |
| P11798 | CAMK2A | 10 | 18.314 |
| P12960 | CNTN1 | 15 | 5.179 |
| P13020 | GSN | 9 | 43.933 |
| P13595 | NCAM1 | 8 | 5.861 |
| P14094 | ATP1B1 | 9 | 7.996 |
| P14152 | MDH1 | 5 | 5.451 |
| P14873 | MAP1B | 33 | 5.209 |
| P15116 | CDH2 | 6 | 21.977 |
| P15508 | SPTB | 33 | 15.339 |
| P16054 | PRKCE | 4 | 5.601 |
| P16330 | CNP | 21 | 7.762 |
| P16546 | SPTAN1 | 178 | 67.831 |
| P16858 | GAPDH | 10 | 6.966 |
| P17156 | HSPA2 | 4 | 6.585 |
| P17426 | AP2A1 | 12 | 12.243 |
| P17427 | AP2A2 | 9 | 6.868 |
| P17710 | HK1 | 7 | 8.681 |
| P18872 | GNAO1 | 9 | 5.486 |
| P20029 | HSPA5 | 23 | 15.662 |
| P20357 | MAP2 | 37 | 7.267 |
| P21107 | TPM3 | 5 | 31.216 |
| P23242 | GJA1 | 14 | 49.73 |
| P23818 | GRIA1 | 6 | 9.597 |
| P23819 | GRIA2 | 6 | 8.561 |
| P27546 | MAP4 | 4 | 8.913 |
| P28652 | CAMK2B | 10 | 20.583 |
| P28660 | NCKAP1 | 6 | 7.493 |
| P28663 | NAPB | 6 | 4.668 |
| P28738 | KIF5C | 8 | 5.319 |
| P28740 | KIF2A | 5 | 35.175 |
| P31648 | SLC6A1 | 4 | 9.315 |
| P31650 | SLC6A11 | 6 | 6.081 |
| P35438 | GRIN1 | 7 | 26.81 |
| P35486 | PDHA1 | 5 | 11.583 |
| P35564 | CANX | 7 | 12.514 |
| P35802 | GPM6A | 7 | 4.762 |
| P38647 | HSPA9 | 16 | 6.337 |
| P39053 | DNM1 | 16 | 5.565 |
| P39447 | TJP1 | 12 | 9.752 |
| P43006 | SLC1A2 | 8 | 12.316 |
| P46096 | SYT1 | 6 | 11.44 |
| P46460 | NSF | 31 | 17.392 |
| P47708 | RPH3A | 5 | 11.816 |
| P47753 | CAPZA1 | 5 | 22.981 |
| P47754 | CAPZA2 | 12 | 36.637 |
| P47757 | CAPZB | 11 | 26.43 |
| P47809 | MAP2K4 | 4 | 14.052 |
| P47857 | PFKM | 4 | 7.679 |
| P48193 | EPB41 | 8 | 9.153 |
| P48722 | HSPA4L | 5 | 6.491 |
| P49813 | TMOD1 | 6 | 7.099 |
| P50446 | KRT6A | 5 | 4.564 |
| P50516 | ATP6V1A | 11 | 24.707 |
| P51863 | ATP6V0D1 | 7 | 11.652 |
| P56399 | USP5 | 6 | 4.733 |
| P57780 | ACTN4 | 22 | 27.393 |
| P58404 | STRN4 | 6 | 27.182 |
| P59764 | DOCK4 | 12 | 4.448 |
| P59999 | ARPC4 | 5 | 21.521 |
| P60201 | PLP1 | 5 | 7.611 |
| P60335 | PCBP1 | 4 | 4.96 |
| P60469 | PPFIA3 | 9 | 6.396 |
| P60521 | GABARAPL2 | 4 | 5.739 |
| P60879 | SNAP25 | 5 | 8.401 |
| P61161 | ACTR2 | 7 | 50.565 |
| P61264 | STX1B | 10 | 5.812 |
| P61979 | HNRNPK | 8 | 6.72 |
| P61982 | YWHAG | 5 | 6.297 |
| P62141 | PPP1CB | 4 | 15.77 |
| P62814 | ATP6V1B2 | 12 | 7.636 |
| P62874 | GNB1 | 5 | 4.816 |
| P62881 | GNB5 | 4 | 10.362 |
| P62984 | UBA52 | 4 | 4.923 |
| P63085 | MAPK1 | 6 | 5.675 |
| P63102 | YWHAZ | 6 | 5.323 |
| P63242 | EIF5A | 5 | 29.71 |
| P63318 | PRKCG | 21 | 24.985 |
| P63328 | PPP3CA | 5 | 5.946 |
| P68254 | YWHAQ | 4 | 7.209 |
| P68368 | TUBA4A | 4 | 18.499 |
| P68404 | PRKCB | 5 | 6.303 |
| P68510 | YWHAH | 5 | 5.403 |
| P70175 | DLG3 | 4 | 100 |
| P70398 | USP9X | 12 | 6.219 |
| P70704 | ATP8A1 | 17 | 16.405 |
| P80560 | PTPRN2 | 15 | 41.09 |
| P84091 | AP2M1 | 6 | 7.636 |
| P97300 | NPTN | 7 | 8.552 |
| P97427 | CRMP1 | 4 | 6.915 |
| P97467 | PAM | 6 | 9.09 |
| P97799 | NRSN1 | 5 | 100 |
| P99024 | TUBB5 | 4 | 8.15 |
| P99029 | PRDX5 | 6 | 6.492 |
| Q00493 | CPE | 6 | 100 |
| Q01405 | SEC23A | 9 | 17.761 |
| Q02248 | CTNNB1 | 8 | 9.343 |
| Q02357 | ANK1 | 5 | 7.235 |
| Q03137 | EPHA4 | 6 | 11.519 |
| Q03141 | MARK3 | 4 | 100 |
| Q03265 | ATP5A1 | 11 | 4.764 |
| Q03517 | SCG2 | 11 | 4.961 |
| Q04690 | NF1 | 5 | 7.315 |
| Q05512 | MARK2 | 8 | 10.815 |
| Q06890 | CLU | 4 | 15.907 |
| Q09200 | B4GALNT1 | 4 | 100 |
| Q0VGU4 | VGF | 22 | 66.374 |
| Q2M3X8 | PHACTR1 | 4 | 100 |
| Q2PFD7 | PSD3 | 11 | 8.961 |
| Q3TXX4 | SLC17A7 | 4 | 5.55 |
| Q3U0V1 | KHSRP | 6 | 4.912 |
| Q3UHB8 | CCDC177 | 6 | 41.278 |
| Q3UHD9 | AGAP2 | 11 | 24.002 |
| Q3UHJ0 | AAK1 | 6 | 4.104 |
| Q3UHK1 | SLC2A13 | 5 | 100 |
| Q3UHL1 | CAMKV | 11 | 9.949 |
| Q3UMT1 | PPP1R12C | 6 | 6.718 |
| Q3UNH4 | GPRIN1 | 5 | 5.736 |
| Q501J6 | DDX17 | 5 | 20.728 |
| Q5DTL9 | SLC4A10 | 4 | 100 |
| Q5PR69 | CRACD | 6 | 100 |
| Q5RJI5 | BRSK1 | 9 | 24.806 |
| Q5SQX6 | CYFIP2 | 5 | 7.066 |
| Q5SXY1 | SPECC1 | 8 | 100 |
| Q60598 | CTTN | 16 | 54.469 |
| Q60605 | MYL6 | 6 | 26.866 |
| Q60625 | ICAM5 | 4 | 5.966 |
| Q60673 | PTPRN | 22 | 100 |
| Q61301 | CTNNA2 | 10 | 16.276 |
| Q61316 | HSPA4 | 4 | 4.082 |
| Q61548 | SNAP91 | 6 | 4.96 |
| Q61553 | FSCN1 | 6 | 14.581 |
| Q61656 | DDX5 | 9 | 12.36 |
| Q61699 | HSPH1 | 9 | 7.382 |
| Q61879 | MYH10 | 82 | 75.182 |
| Q62108 | DLG4 | 8 | 100 |
| Q62188 | DPYSL3 | 4 | 10.5 |
| Q62261 | SPTBN1 | 113 | 88.023 |
| Q62318 | TRIM28 | 4 | 10.761 |
| Q62417 | SORBS1 | 4 | 5.308 |
| Q62420 | SH3GL2 | 4 | 5.808 |
| Q62443 | NPTX1 | 5 | 100 |
| Q641P0 | ACTR3B | 7 | 39.894 |
| Q64331 | MYO6 | 23 | 37.937 |
| Q64332 | SYN2 | 9 | 8.945 |
| Q64521 | GPD2 | 12 | 8.668 |
| Q68FD5 | CLTC | 35 | 13.459 |
| Q68FF6 | GIT1 | 6 | 9.349 |
| Q68FH0 | PKP4 | 13 | 4.539 |
| Q6IFX2 | KRT42 | 4 | 4.312 |
| Q6NS52 | DGKB | 6 | 100 |
| Q6NZL0 | SOGA3 | 6 | 10.551 |
| Q6P1F6 | PPP2R2A | 4 | 23.51 |
| Q6P9K8 | CASKIN1 | 19 | 4.452 |
| Q6PAJ1 | BCR | 5 | 6.444 |
| Q6PGN3 | DCLK2 | 10 | 13.987 |
| Q6PIC6 | ATP1A3 | 18 | 5.179 |
| Q6PIE5 | ATP1A2 | 9 | 4.847 |
| Q6R891 | PPP1R9B | 13 | 45.329 |
| Q6URW6 | MYH14 | 12 | 29.953 |
| Q6ZPE2 | SBF1 | 11 | 10.077 |
| Q6ZPQ6 | PITPNM2 | 4 | 15.555 |
| Q76MZ3 | PPP2R1A | 5 | 12.773 |
| Q7TME0 | PLPPR4 | 7 | 7.964 |
| Q7TNM2 | TRIM46 | 5 | 100 |
| Q7TPH6 | MYCBP2 | 6 | 5.476 |
| Q7TPR4 | ACTN1 | 15 | 58.697 |
| Q7TQF7 | AMPH | 5 | 5.568 |
| Q7TSJ2 | MAP6 | 25 | 21.078 |
| Q80TE7 | LRRC7 | 6 | 100 |
| Q80TR1 | ADGRL1 | 6 | 4.801 |
| Q80TS3 | ADGRL3 | 4 | 4.811 |
| Q80TZ3 | DNAJC6 | 6 | 7.563 |
| Q80UG2 | PLXNA4 | 5 | 5.211 |
| Q80UG5 | SEPTIN9 | 4 | 9.329 |
| Q80WM4 | HAPLN4 | 6 | 25.463 |
| Q80YA9 | CNKSR2 | 8 | 16.35 |
| Q812A2 | SRGAP3 | 4 | 6.631 |
| Q8BFR5 | TUFM | 8 | 14.757 |
| Q8BFZ3 | ACTBL2 | 4 | 80.839 |
| Q8BG39 | SV2B | 4 | 15.492 |
| Q8BG95 | PPP1R12B | 5 | 6.416 |
| Q8BH44 | CORO2B | 11 | 53.982 |
| Q8BHL5 | ELMO2 | 7 | 6.36 |
| Q8BIZ1 | ANKS1B | 5 | 100 |
| Q8BJH1 | ZC2HC1A | 8 | 7.199 |
| Q8BL65 | ABLIM2 | 9 | 100 |
| Q8BLK3 | LSAMP | 4 | 9.936 |
| Q8BMS1 | HADHA | 6 | 5.608 |
| Q8BPN8 | DMXL2 | 17 | 7.255 |
| Q8BPQ7 | SGSM1 | 11 | 49.708 |
| Q8BRT1 | CLASP2 | 13 | 19.395 |
| Q8BTM8 | FLNA | 7 | 7.537 |
| Q8BXR1 | SLC7A14 | 4 | 78.168 |
| Q8C0T5 | SIPA1L1 | 8 | 100 |
| Q8C1B7 | SEPTIN11 | 4 | 13.102 |
| Q8C5W0 | CLMN | 5 | 100 |
| Q8C8R3 | ANK2 | 92 | 37.977 |
| Q8CAQ8 | IMMT | 4 | 7.008 |
| Q8CFI0 | NEDD4L | 4 | 15.422 |
| Q8CHC4 | SYNJ1 | 16 | 5.043 |
| Q8K0S0 | PHYHIP | 5 | 6.962 |
| Q8K0T0 | RTN1 | 8 | 6.425 |
| Q8K0U4 | HSPA12A | 10 | 18.984 |
| Q8K1M6 | DNM1L | 11 | 5.963 |
| Q8K212 | PACS1 | 4 | 100 |
| Q8K310 | MATR3 | 4 | 6.193 |
| Q8K400 | STXBP5 | 5 | 8.21 |
| Q8K406 | LGI3 | 6 | 17.524 |
| Q8K4G5 | ABLIM1 | 8 | 100 |
| Q8K596 | SLC8A2 | 5 | 6.905 |
| Q8QZY9 | SF3B4 | 4 | 100 |
| Q8R071 | ITPKA | 5 | 18.354 |
| Q8R0S2 | IQSEC1 | 7 | 10.653 |
| Q8R366 | IGSF8 | 4 | 10.175 |
| Q8R4U7 | LUZP1 | 14 | 9.586 |
| Q8R5H6 | WASF1 | 5 | 9.566 |
| Q8VD37 | SGIP1 | 5 | 11.169 |
| Q8VDD5 | MYH9 | 48 | 32.66 |
| Q8VDM4 | PSMD2 | 5 | 5.001 |
| Q8VDQ8 | SIRT2 | 4 | 5.224 |
| Q8VEK3 | HNRNPU | 6 | 6.853 |
| Q8VHH5 | AGAP3 | 6 | 12.975 |
| Q8VHW2 | CACNG8 | 4 | 9.882 |
| Q91V41 | RAB14 | 5 | 4.683 |
| Q91VK4 | ITM2C | 5 | 100 |
| Q91VR5 | DDX1 | 7 | 13.848 |
| Q91WC3 | ACSL6 | 4 | 100 |
| Q91WG7 | DGKG | 5 | 100 |
| Q91YR1 | TWF1 | 5 | 11.703 |
| Q91Z67 | SRGAP2 | 13 | 13.868 |
| Q91ZU6 | DST | 8 | 6.227 |
| Q920I9 | WDR7 | 10 | 8.025 |
| Q922R8 | PDIA6 | 6 | 5.85 |
| Q922S4 | PDE2A | 5 | 6.366 |
| Q99104 | MYO5A | 4 | 56.383 |
| Q99J85 | NPTXR | 8 | 55.983 |
| Q99JY9 | ACTR3 | 9 | 29.515 |
| Q99K51 | PLS3 | 5 | 7.451 |
| Q99KI0 | ACO2 | 6 | 8.262 |
| Q99KJ8 | DCTN2 | 4 | 4.574 |
| Q99M73 | KRT84 | 7 | 100 |
| Q99M87 | DNAJA3 | 4 | 18.985 |
| Q99P72 | RTN4 | 5 | 4.848 |
| Q9CVB6 | ARPC2 | 8 | 16.759 |
| Q9CWZ7 | NAPG | 5 | 6.689 |
| Q9CZU6 | CS | 4 | 24.841 |
| Q9D0E1 | HNRNPM | 5 | 25.842 |
| Q9D1T0 | LINGO1 | 6 | 11.427 |
| Q9D394 | RUFY3 | 4 | 6.705 |
| Q9D6R2 | IDH3A | 6 | 4.48 |
| Q9D8Y0 | EFHD2 | 11 | 7.376 |
| Q9DBG3 | AP2B1 | 6 | 7.421 |
| Q9DBH5 | LMAN2 | 4 | 24.007 |
| Q9DBR7 | PPP1R12A | 12 | 35.829 |
| Q9EPN1 | NBEA | 14 | 5.714 |
| Q9ERD7 | TUBB3 | 6 | 22.557 |
| Q9ESJ4 | NCKIPSD | 9 | 16.99 |
| Q9JHU4 | DYNC1H1 | 30 | 4.287 |
| Q9JIA1 | LGI1 | 6 | 10.603 |
| Q9JIS5 | SV2A | 7 | 12.265 |
| Q9JJ28 | FLII | 13 | 25.379 |
| Q9JJK2 | LANCL2 | 4 | 6.284 |
| Q9JJV2 | PFN2 | 4 | 8.949 |
| Q9JKC6 | CEND1 | 10 | 12.315 |
| Q9JKK7 | TMOD2 | 10 | 65.399 |
| Q9JLM8 | DCLK1 | 16 | 21.984 |
| Q9JLN9 | MTOR | 7 | 9.757 |
| Q9JM52 | MINK1 | 8 | 8.043 |
| Q9JMH9 | MYO18A | 48 | 44.339 |
| Q9QWI6 | SRCIN1 | 31 | 16.8 |
| Q9QXS1 | PLEC | 55 | 14.95 |
| Q9QXS6 | DBN1 | 34 | 92.988 |
| Q9QXV0 | PCSK1N | 4 | 19.304 |
| Q9QXZ0 | MACF1 | 33 | 5.947 |
| Q9QY76 | VAPB | 4 | 5.175 |
| Q9QYB5 | ADD3 | 9 | 5.968 |
| Q9QYB8 | ADD2 | 11 | 36.336 |
| Q9QYC0 | ADD1 | 17 | 39.338 |
| Q9QYJ0 | DNAJA2 | 4 | 16.554 |
| Q9QYR6 | MAP1A | 39 | 4.014 |
| Q9QYX7 | PCLO | 19 | 4.678 |
| Q9QZX7 | SRR | 5 | 4.986 |
| Q9R0K7 | ATP2B2 | 7 | 8.025 |
| Q9R0Q6 | ARPC1A | 9 | 55.779 |
| Q9R111 | GDA | 6 | 11.391 |
| Q9R1Q8 | TAGLN3 | 8 | 45.28 |
| Q9R1R2 | TRIM3 | 7 | 19.464 |
| Q9WTI7 | MYO1C | 5 | 11.937 |
| Q9WTM5 | RUVBL2 | 4 | 6.101 |
| Q9WUM4 | CORO1C | 7 | 100 |
| Q9WV69 | DMTN | 10 | 72.713 |
| Q9WV92 | EPB41L3 | 13 | 9.514 |
| Q9Z0E0 | NCDN | 13 | 17.232 |
| Q9Z0H8 | CLIP2 | 10 | 13.039 |
| Q9Z0P4 | PALM | 5 | 7.096 |
| Q9Z0P5 | TWF2 | 5 | 4.915 |
| Q9Z0U1 | TJP2 | 9 | 7.339 |
| Q9Z140 | CPNE6 | 10 | 9.15 |
| Q9Z1G3 | ATP6V1C1 | 4 | 10.217 |
| Q9Z1G4 | ATP6V0A1 | 13 | 11.659 |
| Q9Z218 | DPP6 | 7 | 6.059 |
| Q9Z2I0 | LETM1 | 5 | 5.712 |
| Q9Z2I9 | SUCLA2 | 7 | 6.988 |
| Q9Z2Q6 | SEPTIN5 | 6 | 7.136 |
| Q9Z2Y3 | HOMER1 | 4 | 12.937 |

**Table S7. GO enrichment analysis of PTPRN interactors**

| Category | Term | Count | *p* value | Gene Symbol |
| --- | --- | --- | --- | --- |
| Biological Process | Actin cytoskeleton organization | 32 | 6.09E-21 | ACTN1, SPTB, MYH10, SPTBN1, FLNA, BCR, ITPKA, EPB41, CTTNBP2, FSCN1, ACTR2, PPP1R9B, IQSEC1, CAPZA1, CAPZA2, PFN2, CAPZB, CORO2B, AGAP2, CORO1C, WASF1, SPECC1, PLEC, SPTAN1, NF1, DMTN, FLII, GSN, SPTA1, ACTN4, PHACTR1, ABLIM2 |
| Biological Process | Nervous system development | 26 | 2.01E-07 | BRSK1, KIF2A, NPTN, GAP43, SIRT2, CRMP1, DPYSL3, SHANK1, MAP1B, DPYSL2, PLXNA4, PPP1R9B, SRGAP2, GPM6A, CAMK2B, RUFY3, NRSN1, IMPACT, DCLK1, RTN4, CNTN1, DBN1, EPHA4, CTNNB1, SCN2A, SLC1A2 |
| Biological Process | Ion transport | 25 | 1.31E-04 | CACNG8, SLC4A10, ATP1A3, ATP6V1A, SLC17A7, ATP6V0D1, GRIN1, SLC4A1, CACNA2D1, ATP1A2, ATP2B1, ATP6V1B2, SLC8A2, RYR2, LETM1, ADD2, ATP6V0A1, ATP5F1A, ATP6V1C1, GRIA1, GRIA2, ATP2A2, ATP2B2, ATP1B1, SCN2A |
| Biological Process | Phosphorylation | 25 | 1.41E-04 | BRSK1, BCR, MTOR, ITPKA, DGKI, MAPK1, AAK1, MINK1, PFKM, CAMK2B, PIP5K1C, CAMK2A, DCLK1, DGKG, MARK3, HK1, PRKCB, DGKB, MAP2K4, PRKCE, EPHA4, MARK2, DCLK2, PRKCG, PI4KA |
| Biological Process | Protein transport | 25 | 3.47E-04 | NAPB, TRIM3, LMAN2, AP2A2, AP2A1, AP2M1, ANK2, MYO5A, MYH9, SEC23A, STXBP1, STX1B, EIF5A, SV2B, STXBP5, NAPG, SNAP91, NSF, AP2B1, MYO6, ACTN4, RPH3A, MYO1C, RAB14, GABARAPL2 |
| Biological Process | Microtubule cytoskeleton organization | 22 | 1.91E-13 | DST, TUBB5, TRIM46, KIF2A, MAPT, GAPDH, MAP6, CRMP1, MAP7D1, MARK3, MAP1B, TUBB3, CNP, MARK2, TUBA4A, MAP1A, DCLK2, CLASP2, NEFL, MAP4, MAP2, TUBA1B |
| Biological Process | Neuron projection development | 20 | 2.03E-10 | NPTXR, GPM6A, MYH10, MAPT, GPRIN1, LGI1, LINGO1, CYFIP2, WASF1, NCDN, SH3GL2, GNAO1, CNTN1, CNTNAP1, NCAM1, PPP1R9B, CAPZB, MAP4, MAP2, CLMN |
| Biological Process | Brain development | 20 | 2.46E-07 | SYT1, MYH10, SLC6A11, BCR, MTOR, DCLK1, SLC17A7, ATP6V0D1, CDH2, NF1, CTTNBP2, GIT1, CNTN1, ADGRL3, SYNJ1, SRR, DPYSL2, MINK1, PPP3CA, ATP2B1 |
| Biological Process | Axonogenesis | 18 | 3.19E-12 | CTNNA2, STXBP5, DST, BRSK1, TRIM46, SNAP91, MYH10, MAPT, ACTBL2, PIP5K1C, DCLK1, PLPPR4, MAP1B, CNTNAP1, CNP, MAP1A, ANK3, MAP2 |
| Biological Process | Synapse organization | 17 | 4.97E-14 | PTPRS, NPTN, SLC6A1, MAPT, FLNA, SNCA, PCLO, ADGRL3, SYN1, BSN, MYO5A, ATP2B2, CTNNB1, ANK3, CTNND2, SLC8A2, PPFIA3 |

**Table S8. KEGG enrichment analysis of PTPRN interactors**

| Category | Term | Count | *p* value | Gene Symbol |
| --- | --- | --- | --- | --- |
| Pathway | Pathways of neurodegeneration - multiple diseases | 30 | 9.73E-07 | TUBB5, DLG4, MAPT, PSMD2, MTOR, GRIN1, SNCA, VAPB, TUBB3, MAPK1, PPP3CA, KIF5C, DCTN2, RYR2, TUBA1B, SEPTIN5, CAMK2B, ATP5F1A, CAMK2A, PRKCB, GRIA1, GRIA2, ATP2A2, HSPA5, TUBA4A, CTNNB1, PRKCG, CALM2, NEFL, UBA52 |
| Pathway | Endocytosis | 28 | 9.92E-11 | CLTC, SH3GL2, AP2A2, AP2A1, AP2M1, GIT1, ACTR3, ACTR2, AMPH, HSPA2, AGAP3, ARPC2, IQSEC1, CAPZA1, KIF5C, CAPZA2, NEDD4L, CAPZB, DNAJC6, AGAP2, PIP5K1C, AP2B1, BIN1, ARPC1A, ACTR3B, ARPC4, PSD3, DNM1 |
| Pathway | Tight junction | 24 | 3.33E-12 | PPP2R2A, CTTN, ACTN1, HSPA4, MYH10, DLG3, MYH11, ARPC1A, ACTR3B, TJP1, ARPC4, ACTR3, PPP2R1A, ACTR2, MYH9, ARPC2, TUBA4A, PRKCE, ACTN4, MYL6, MYH14, NEDD4L, TJP2, TUBA1B |
| Pathway | Regulation of actin cytoskeleton | 24 | 9.74E-10 | ACTN1, NCKAP1, PPP1R12B, MYH10, PIP5K1C, MYH11, CYFIP2, ARPC1A, ACTR3B, WASF1, ARPC4, GIT1, ACTR3, PPP1R12A, GSN, ACTR2, MYH9, ARPC2, MAPK1, ACTN4, PPP1CB, PPP1R12C, MYH14, PFN2 |
| Pathway | Salmonella infection | 24 | 1.47E-08 | HSP90AA1, TUBB5, NCKAP1, FLNA, GAPDH, CYFIP2, ARPC1A, MYO6, ACTR3B, ARPC4, ACTR3, ACTR2, ARPC2, TUBB3, MAPK1, MAP2K4, TUBA4A, CTNNB1, KIF5C, ELMO2, PFN2, DYNC1H1, DCTN2, TUBA1B |
| Pathway | Synaptic vesicle cycle | 20 | 4.85E-15 | SYT1, SNAP25, SLC6A1, NSF, CLTC, ATP6V0A1, AP2B1, SLC6A11, ATP6V1A, SLC17A7, ATP6V0D1, AP2A2, AP2A1, AP2M1, ATP6V1C1, STXBP1, DNM1, STX1B, ATP6V1B2, SLC1A2 |
| Pathway | Adrenergic signaling in cardiomyocytes | 18 | 6.20E-08 | PPP2R2A, CACNG8, CAMK2B, ATP1A3, CAMK2A, PPP2R1A, CACNA2D1, TPM3, ATP2A2, ATP2B2, MAPK1, PPP1CB, ATP1B1, ATP1A2, CALM2, ATP2B1, SLC8A2, RYR2 |
| Pathway | Proteoglycans in cancer | 17 | 1.82E-05 | CTTN, CAMK2B, DDX5, PPP1R12B, FLNA, CAMK2A, MTOR, PPP1R12A, PRKCB, ANK2, MAPK1, PPP1CB, ANK1, CTNNB1, PPP1R12C, ANK3, PRKCG |
| Pathway | Oxytocin signaling pathway | 16 | 2.09E-06 | CACNG8, CAMK2B, PPP1R12B, CAMK2A, GNAO1, CACNA2D1, PPP1R12A, PRKCB, MAPK1, PPP1CB, PPP1R12C, PPP3CA, MYL6, PRKCG, CALM2, RYR2 |
| Pathway | Bacterial invasion of epithelial cells | 15 | 1.40E-09 | CTNNA2, CTTN, SEPTIN11, CLTC, ARPC1A, ACTR3B, WASF1, ARPC4, ACTR3, ACTR2, ARPC2, DNM1, CTNNB1, ELMO2, SEPTIN9 |

**Table S9. Properties of Na_V_1.2-mediated currents in HEK-293T cells in the presence of PTPRN**

| Condition | Peak current density (pA/pF) | Activation V_1/2_ (mV) | Inactivation V_1/2_ (mV) | Recovery time constant τ (s^-1^) |
| --- | --- | --- | --- | --- |
| Na_V_1.2 (n=18) | -96.17 ± 11.8 | -19.21 ± 0.89 | -52.31 ± 0.81 | 1.94 ± 0.09 |
| Na_V_1.2 + PTPRN (1:1, n=18) | -78.45 ± 11.9* | -14.89 ± 1.04* | -52.47 ± 0.88 | 1.91 ± 0.17 |
| Na_V_1.2 + PTPRN (1:2, n=20) | -56.89 ± 7.59* | -11.63 ± 1.26**** | -51.94 ± 0.91 | 1.69 ± 0.11 |

Data are reported as mean ± s.e.m. for cells expressing Na_V_1.2 channels. **p* < 0.05, *****p* < 0.0001, one-way ANOVA with Bonferroni’ s multiple-comparison test.

**Table S10. Properties of Na_V_1.5-mediated currents in HEK-293T cells in the presence of PTPRN**

| Condition | Peak current density (pA/pF) | Activation V_1/2_ (mV) | Inactivation V_1/2_ (mV) | Recovery time constant τ (s^-1^) |
| --- | --- | --- | --- | --- |
| Na_V_1.5 (n=19) | -427.1 ± 31.89 | -37.86 ± 1.08 | -68.82 ± 1.68 | 2.06 ± 0.12 |
| Na_V_1.5 + PTPRN (n=19) | -391.8 ± 37.61 | -37.92 ± 0.89 | -68.47 ± 1.36 | 1.96 ± 0.09 |

Data are reported as mean ± s.e.m. for cells expressing Na_V_1.5 channels. Unpaired two-tailed Student’s t-test.

**Table S11. Properties of Na_V_1.6-mediated currents in HEK-293 cells in the presence of PTPRN**

| Condition | Peak current density (pA/pF) | Activation V_1/2_ (mV) | Inactivation V_1/2_ (mV) | Recovery time constant τ (s^-1^) |
| --- | --- | --- | --- | --- |
| Na_V_1.6 (n=21) | -105.6 ± 24.90 | -18.89 ± 0.78 | -54.00 ± 0.68 | 2.26 ± 0.15 |
| Na_V_1.6 + PTPRN (n=17) | -101.5 ± 16.78 | -18.74 ± 0.65 | -53.33 ± 0.56 | 2.12 ± 0.12 |

Data are reported as mean ± s.e.m. for cells expressing Na_V_1.6 channels. Unpaired two-tailed Student’s t-test.

**Table S12. Properties of I_Na_ in primary neurons isolated from PTPRN-WT/KO mice**

| Condition | Peak current density (pA/pF) |
| --- | --- |
| PTPRN-WT (n=13) | -353.3 ± 31.64 |
| PTPRN-KO (n=16) | -617.0 ± 66.77** |

Data are reported as mean ± s.e.m. for primary neurons. ***p* < 0.01, unpaired two-tailed Student’s t-test.

**Table S13. Properties of I_Na_ in primary neurons isolated from Scn8a-KO mice**

| Condition | Peak current density (pA/pF) |
| --- | --- |
| nonsilencing-shRNA (n=15) | -453.8 ± 67.27 |
| shRNA-PTPRN#3 (n=15) | -814.2 ± 91.33** |

Data are reported as mean ± s.e.m. for primary neurons. ***p* < 0.01, unpaired two-tailed Student’s t-test.

**Table S14. Properties of Na_V_1.2-mediated currents in HEK-293T cells treated with Dynasore**

| Condition | Peak current density (pA/pF) | Activation V_1/2_ (mV) |
| --- | --- | --- |
| Na_V_1.2 (n=21) | -110.7 ± 11.7 | -16.42 ± 0.62 |
| Na_V_1.2 + PTPRN (n=21) | -73.40 ± 7.12 | -9.151 ± 0.88 |
| Na_V_1.2 + Dynasore (n=21) | -118.2 ± 7.38 | -10.32 ± 0.85 |
| Na_V_1.2 + PTPRN + Dynasore (n=24) | -109.8 ± 9.44 | -8.970 ± 0.87 |

Data are reported as mean ± s.e.m. for cells expressing Na_V_1.2 channels.

**Table S15. Properties of Na_V_1.2-mediated currents in HEK-293T cells treated with BFA**

| Condition | Peak current density (pA/pF) | Activation V_1/2_ (mV) |
| --- | --- | --- |
| Na_V_1.2 (n=18) | -108.0 ± 10.2 | -17.24 ± 0.37 |
| Na_V_1.2 + PTPRN (n=26) | -77.64 ± 6.90 | -10.96 ± -1.1 |
| Na_V_1.2 + BFA (n=20) | -65.57 ± 8.16 | -11.85 ± 0.69 |
| Na_V_1.2 + PTPRN + BFA (n=21) | -35.77 ± 3.46 | -8.280 ± 0.80 |

Data are reported as mean ± s.e.m. for cells expressing Na_V_1.2 channels.

**Table S16. Properties of Na_V_1.2-mediated currents in HEK-293T cells treated with Pitstop2**

| Condition | Peak current density (pA/pF) | Activation V_1/2_ (mV) |
| --- | --- | --- |
| Na_V_1.2 (n=18) | -108.0 ± 10.2 | -17.24 ± 0.37 |
| Na_V_1.2 + PTPRN (n=26) | -77.64 ± 6.90 | -10.96 ± -1.1 |
| Na_V_1.2 + Pitstop2 (n=26) | -130.3 ± 10.4 | -17.74 ± 0.30 |
| Na_V_1.2 + PTPRN + Pitstop2 (n=24) | -109.0 ± 11.7 | -14.96 ± 0.66 |

Data are reported as mean ± s.e.m. for cells expressing Na_V_1.2 channels. The data for the Na_V_1.2 group and the Na_V_1.2 + PTPRN group are equivalent to the data in Table S15.

**Table S17. Properties of Na_V_1.2-mediated currents in HEK-293T cells treated with TAK243**

| Condition | Peak current density (pA/pF) | Activation V_1/2_ (mV) |
| --- | --- | --- |
| Na_V_1.2 (n=18) | -108.0 ± 10.2 | -17.24 ± 0.37 |
| Na_V_1.2 + PTPRN (n=26) | -77.64 ± 6.90 | -10.96 ± -1.1 |
| Na_V_1.2 + TAK243 (n=20) | -115.6 ± 10.9 | -14.78 ± 0.35 |
| Na_V_1.2 + PTPRN + TAK243 (n=26) | -115.7 ± 12.1 | -16.16 ± 0.74 |

Data are reported as mean ± s.e.m. for cells expressing Na_V_1.2 channels. The data for the Na_V_1.2 group and the Na_V_1.2 + PTPRN group are equivalent to the data in Table S15.

**Table S18. Properties of Na_V_1.2-mediated currents in HEK-293T cells treated with Heclin**

| Condition | Peak current density (pA/pF) | Activation V_1/2_ (mV) |
| --- | --- | --- |
| Na_V_1.2 (n=18) | -108.0 ± 10.2 | -17.24 ± 0.37 |
| Na_V_1.2 + PTPRN (n=26) | -77.64 ± 6.90 | -10.96 ± -1.1 |
| Na_V_1.2 + Heclin (n=19) | -116.9 ± 12.1 | -16.13 ± 0.51 |
| Na_V_1.2 + PTPRN + Heclin (n=19) | -119.2 ± 13.5 | -17.65 ± 0.62 |

Data are reported as mean ± s.e.m. for cells expressing Na_V_1.2 channels. The data for the Na_V_1.2 group and the Na_V_1.2 + PTPRN group are equivalent to the data in Table S15.

**Table S19. Properties of Na_V_1.2-mediated currents in HEK-293T cells with the equilibration of purified peptide**

| Condition | Peak current density (pA/pF) | Activation V_1/2_ (mV) |
| --- | --- | --- |
| Na_V_1.2 + TagTFP (n=25) | -116.1 ± 11.6 | -25.60 ± 0.73 |
| Na_V_1.2 + PTPRN-cyto (n=23) | -122.1 ± 10.1 | -25.83 ± 0.90 |

Data are reported as mean ± s.e.m. for cells expressing Na_V_1.2 channels. Unpaired two-tailed Student’s t-test.

**Table S20. Properties of I_Na_ in HEK-293T cells in the presence of PTPRN**

| Condition | Peak current density (pA/pF) | Activation V_1/2_ (mV) | Inactivation V_1/2_ (mV) |
| --- | --- | --- | --- |
| Na_V_1.2 (n=21) | -116.5 ± 12.1 | -16.56 ± 0.64 | -52.31 ± 0.76 |
| Na_V_1.5 (n=19) | -427.1 ± 31.9 | -38.84 ± 0.78 | -68.82 ± 1.6 |
| Na_V_1.2/5C (n=19) | -114.6 ± 12.7 | -16.41 ± 0.61 | -43.10 ± 0.70 |
| Na_V_1.5/2C (n=20) | -105.7 ± 11.7 | -37.10 ± 0.93 | -77.05 ± 1.2 |
| Na_V_1.2/5C + PTPRN (n=26) | -136.6 ± 7.98 | -16.69 ± 0.44 | \ |
| Na_V_1.5/2C + PTPRN (n=20) | -56.46 ± 6.67 | -33.91 ± 0.79 | \ |

Data are reported as mean ± s.e.m. for cells expressing Na_V_ channels.

**Table S21. Properties of mutated Na_V_1.2-mediated currents in HEK-293T cells in the presence of PTPRN**

| Condition | Peak current density (pA/pF) | Activation V_1/2_ (mV) |
| --- | --- | --- |
| Na_V_1.2 (n=20) | -111.6 ± 10.32 | -16.05 ± 0.73 |
| Na_V_1.2^IL-AA^ (n=22) | -100.3 ± 7.06 | -16.84 ± 0.73 |
| Na_V_1.2^IL-AA^ + PTPRN (n=23) | -63.69 ± 6.05 | -10.77 ± 0.80 |
| Na_V_1.2 ^PPSY-AAAA^ (n=22) | -101.7 ± 10.3 | -17.58 ± 0.66 |
| Na_V_1.2 ^PPSY-AAAA^ + PTPRN (n=22) | -117.0 ± 8.12 | -16.51 ± 0.67 |

Data are reported as mean ± s.e.m. for cells expressing Na_V_1.2 channels.

**Table S22. Properties of Na_V_1.2-mediated currents in HEK-293T cells in the presence of PTPRN**

| Condition | Peak current density (pA/pF) | Activation V_1/2_ (mV) |
| --- | --- | --- |
| Na_V_1.2 (n=18) | -99.66 ± 12.15 | -18.45 ± 0.85 |
| Na_V_1.2 + PTPRN (n=24) | -78.32 ± 7.26 | -13.09 ± 0.82 |
| Na_V_1.2 + NEDD4L^CS^ (n=24) | -151.3 ± 15.31 | -18.79 ± 0.78 |
| Na_V_1.2 + NEDD4L^CS^ + PTPRN (n=23) | -137.3 ± 9.45 | -18.73 ± 0.75 |

Data are reported as mean ± s.e.m. for cells expressing Na_V_1.2 channels.

**Table S23. Properties of I_Na_ current in primary neurons isolated from SCN8A- KO mice**

| Condition | Peak current density (pA/pF) |
| --- | --- |
| nonsilencing-shRNA + shRNA-Nedd4l (n=15) | -410.5 ± 47.19 |
| shRNA-PTPRN#3 + shRNA-Nedd4l (n=15) | -343.8 ± 31.44 |

Data are reported as mean ± s.e.m. for primary neurons. Unpaired two-tailed Student’s t-test.

**Table S24. Electrophysiological properties of hippocampal DG granule cells of mice**

| Parameters | PTPRN-WT + nonsilencing-shRNA (n=18) | PTPRN-KO + nonsilencing-shRNA (n=26) | PTPRN-KO + shRNA-SCN2A (n=18) |
| --- | --- | --- | --- |
| Input resistance (MΩ) | 223.2 ± 14.10 | 192.6 ± 9.136 | 202.8 ± 14.25 |
| Threshold (mV) | -47.32 ± 0.7003 | -45.92 ± 0.8561 | -45.20 ± 0.6790 |
| Peak dV/dt (mV/ms) | 191.4 ± 6.658 | 214.7 ± 5.284* | 115.7 ± 7.106**** |
| Amplitude (mV) | 121.5 ± 1.036 | 123.2 ± 0.5504 | 107.3 ± 1.286**** |
| Half width (ms) | 1.609 ± 0.0440 | 1.487 ± 0.0360 | 1.443 ± 0.0365 |
| Rise slope (mV/ms) | 143.5 ± 6.439 | 155.4 ± 3.669 | 86.67 ± 5.458**** |
| Decay slope (mV/ms) | -40.33 ± 1.120 | -44.31 ± 1.029* | -40.99 ± 1.340 |

Data are reported as mean ± s.e.m. for DG granule cells of mice. **p* < 0.05, *****p* < 0.0001, one-way ANOVA with Bonferroni’ s multiple-comparison test.

**Table S25. qRT-PCR Primers**

| Gene | Strand | Sequence |
| --- | --- | --- |
| mouse PTPRN | F | ACCAAGGTTGCCAGAGAATGG |
| mouse PTPRN | R | CCTGCCAGTACAGCAGTCAAT |
| mouse β-Actin | F | GGCTGTATTCCCCTCCATCG |
| mouse β-Actin | R | CCAGTTGGTAACAATGCCATGT |
| mouse SCN2A | F | ATTTTCGGCTCATTCTTCACACT |
| mouse SCN2A | R | GGGCGAGGTATCGGTTTTTGT |
| mouse NEDD4L | F | GACATGGAGCATGGATGGGAA |
| mouse NEDD4L | R | GTTCGGCCTAAATTGTCCACT |
| mouse c-Fos | F | ATCCTTGGAGCCAGTCAAGA |
| mouse c-Fos | R | ATGATGCCGGAAACAAGAAG |
| rat PTPRN | F | CCCCAAGCAGTACAAGCAGAT |
| rat PTPRN | R | GTTCACAGCCCCTCCAAGAT |
| rat β-Actin | F | gccgggacctgacagactac |
| rat β-Actin | R | ctgtcagcaatgcctgggtac |
